# Hermansky-Pudlak syndrome type 1 causes impaired anti-microbial immunity and inflammation due to dysregulated immunometabolism

**DOI:** 10.1038/s41385-022-00572-1

**Published:** 2022-10-27

**Authors:** Athena Cavounidis, Sumeet Pandey, Melania Capitani, Matthias Friedrich, Amy Cross, Lisa Gartner, Dominik Aschenbrenner, Seunghee Kim-Schulze, Ying Ka Lam, Georgina Berridge, Dermot P. B. McGovern, Benedikt Kessler, Roman Fischer, Paul Klenerman, Joanna Hester, Fadi Issa, Esther A. Torres, Fiona Powrie, Bernadette R. Gochuico, William A. Gahl, Louis Cohen, Holm H. Uhlig

**Affiliations:** 1 Translational Gastroenterology Unit, Nuffield Department of Medicine, University of Oxford, Oxford, UK; 2 Kennedy Institute of Rheumatology, Nuffield Department of Orthopaedics, Rheumatology and Musculoskeletal Sciences, University of Oxford, Oxford, UK; 3 Nuffield Department of Surgical Sciences, University of Oxford, Oxford, UK; 4 Human Immune Monitoring Center, Icahn School of Medicine at Mount Sinai, New York, NY USA; 5 Target Discovery Institute, Center for Medicines Discovery, Nuffield Department of Medicine, University of Oxford, Oxford, UK; 6 F. Widjaja Foundation Inflammatory Bowel and Immunobiology Institute, Cedars-Sinai Medical Center, Los Angeles, CA USA; 7 University of Puerto Rico School of Medicine, Puerto Rico, USA; 8 Medical Genetics Branch, National Human Genome Research Institute, National Institutes of Health, Bethesda, MD USA; 9 Icahn School of Medicine at Mount Sinai, New York, NY USA; 10 Department of Paediatrics, University of Oxford, Oxford, UK; 11 Oxford NIHR Biomedical Research Centre, Oxford, UK; 12 Present Address: GSK, Wavre, Belgium; 13 Present Address: GSK Immunology Network, GSK Medicines Research Center, Stevenage, UK; 14 Present Address: SenTcell Ltd, London, UK; 15 Present Address: Autoimmunity, Transplantation and Inflammation, Novartis Institutes for BioMedical Research, Novartis Pharma AG, Basel, Switzerland

## Abstract

Hermansky-Pudlak syndrome (HPS) types 1 and 4 are caused by defective vesicle trafficking. The mechanism for Crohn’s disease-like inflammation, lung fibrosis, and macrophage lipid accumulation in these patients remains enigmatic. The aim of this study is to understand the cellular basis of inflammation in HPS-1. We performed mass cytometry, proteomic and transcriptomic analyses to investigate peripheral blood cells and serum of HPS-1 patients. Using spatial transcriptomics, granuloma-associated signatures in the tissue of an HPS-1 patient with granulomatous colitis were dissected. In vitro studies were conducted to investigate anti-microbial responses of HPS-1 patient macrophages and cell lines. Monocytes of HPS-1 patients exhibit an inflammatory phenotype associated with dysregulated TNF, IL-1α, OSM in serum, and monocyte-derived macrophages. Inflammatory macrophages accumulate in the intestine and granuloma-associated macrophages in HPS-1 show transcriptional signatures suggestive of a lipid storage and metabolic defect. We show that HPS1 deficiency leads to an altered metabolic program and Rab32-dependent amplified mTOR signaling, facilitated by the accumulation of mTOR on lysosomes. This pathogenic mechanism translates into aberrant bacterial clearance, which can be rescued with mTORC1 inhibition. Rab32-mediated mTOR signaling acts as an immuno-metabolic checkpoint, adding to the evidence that defective bioenergetics can drive hampered anti-microbial activity and contribute to inflammation.

## Introduction

Understanding the cell biology of rare genetic disorders can provide novel insights into immunological mechanisms that are key for a wider range of immune-mediated disorders. Specifically, it expands our view of inflammatory mechanisms in humans with defined, highly penetrant, pathogenic protein-coding variants. Inflammatory bowel disease (IBD) is increasingly seen as a complex group of disorders. Changes in immunometabolism have been observed but it is not clear whether this is a cause or consequence of intestinal inflammation and requires molecular understanding. Intestinal macrophages in both Crohn’s disease and ulcerative colitis have reduced oxidative phosphorylation and fatty acid degradation^1^. Furthermore, increased levels of lipid droplets have been observed in the dextran sodium sulfate mouse model of colitis^2^. Specifically, prostaglandin E2, which is synthesized by PTGS2, in combination with TNF, mediates an increase in lipid droplets. Lipid droplet metabolism is important in monocyte-to-macrophage differentiation, playing a role in macrophage function^3^. Disorders such as HPS-1 can lend a helping hand in navigating yet-to-be-defined features in IBD, such as metabolic alterations.

Patients with Hermansky-Pudlak syndrome (HPS) present with oculocutaneous albinism, platelet storage pool defects, and ceroid lipofuscin accumulation^4^. HPS is autosomal recessive and can be caused by mutations in eleven different genes^5,6^. A subset of HPS-1 and HPS-4 patients can develop severe, chronic intestinal inflammation and lung fibrosis. The intestinal inflammation and lung fibrosis are reminiscent of Crohn’s disease and idiopathic pulmonary fibrosis, respectively^4,7,8^. The intestinal inflammation can involve fistulae, granulomata and ceroid lipofuscinosis^8^. These clinical manifestations suggest a link between HPS and the more common polygenic counterparts. However, the underlying reason why HPS-1 and HPS-4 patients specifically develop chronic inflammatory diseases remains elusive^4,8,9^.

The HPS1 and HPS4 proteins interact divalently to form Biogenesis of Lysosome-related Organelles Complex 3 (BLOC-3)^10,11^. BLOC-3 acts as a guanine nucleotide exchange factor (GEF) for Rab32 and Rab38^12^. Whereas the function of the BLOC-3 complex for melanosome formation is well established, recent data have begun to indicate a mechanistic role in the immune system^12,13^. A comparison of blood cytokines between HPS-1 patients with pulmonary fibrosis (PF) and patients with familial pulmonary fibrosis revealed a distinct inflammatory signature in HPS-1 with elevation of IFN-γ, TNF and IL-8^14^. Recent data also demonstrate that HPS4 deficiency in iPS cell-derived macrophages or Rab32 deficiency in mice can lead to an inability to combat the pathogen *Salmonella typhi*^15,16^. However, the specific mechanisms that drive intestinal and pulmonary immune dysregulation in HPS-1 patients are not clear. In this study, we aim to showcase the immunological underpinnings of HPS-1 and the defective signaling cascades that contribute to the pathology of the disease.

We performed a multi-omic analysis in patients with HPS-1 to investigate the inflammatory cellular and cytokine landscape. This analysis highlights the importance of the myeloid cell compartment to the immune dysregulation observed in HPS-1 and defines an inflammatory signature in monocytes and macrophages. Functional studies further demonstrate a molecular mechanism through which HPS1 deficiency alters metabolism, results in Rab32-mediated amplified mTOR signaling, and leads to defective bacterial handling and dysregulated cytokine production.

## Results

### Mass cytometry highlights the presence of inflammatory monocytes in patients with HPS-1

**Fig. 1:**
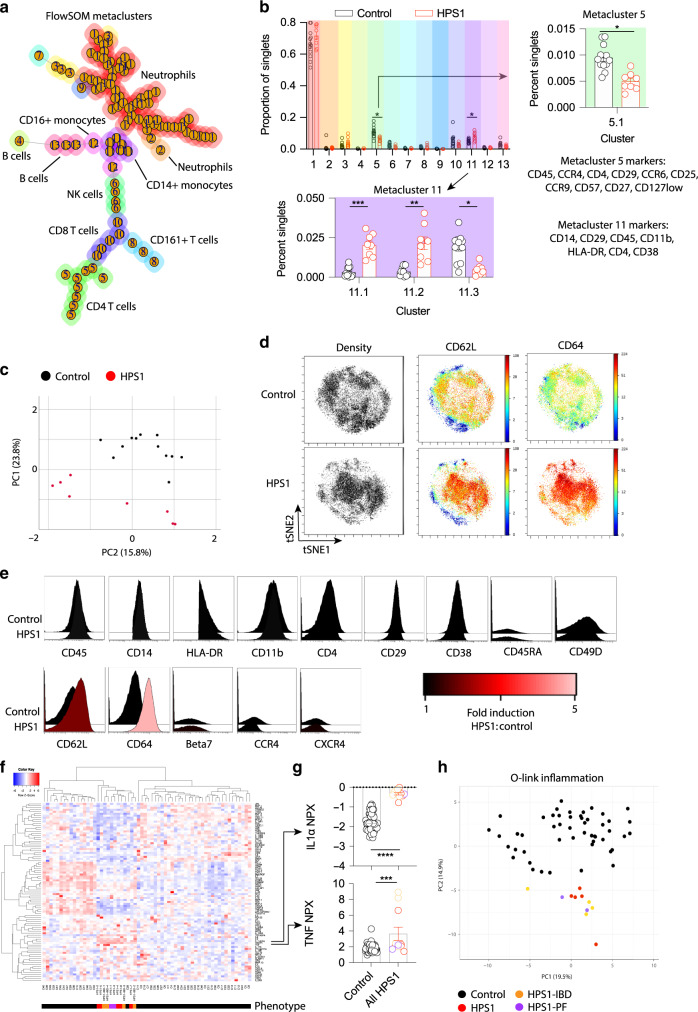
Mass cytometry of HPS-1 PBMCs identifies distinct inflammatory monocyte populations. **a** FlowSOM analysis of CyTOF data defines 100 cell clusters (individual nodes) organized into 13 metaclusters (node number and color) for 12 controls and 8 HPS-1 patients. We denoted clusters with surface markers that resembled particular cell types. **b** Specific metacluster 11 and metacluster 5 cell clusters are significantly associated with HPS-1 patients. Metacluster 11.1 was defined by CXCR3, CD64++, CD62L++, metacluster 11.2 by Beta7++, CD64++, CD62L++ and 11.3 by CD16. **c** Principal component analysis of FlowSOM clusters separates HPS-1 patients and healthy controls. **d** viSNE analysis of CD14^+^ monocytes defines distinct populations of monocytes in patients with HPS-1 highly expressing CD62L and CD64. **e** Histogram of marker expression in patients with HPS-1 or Healthy Controls. **f** Heatmap with hierarchical clustering of inflammatory proteins of O-link inflammation panel for 50 controls, 5 HPS-1, 2 HPS-1 PF, and 4 HPS-1 IBD patients. **g** Dot plots of IL-1α and TNF protein expression. **h** Principal component analysis of the O-link inflammatory panel. For CyTOF analysis, populations are compared with an unpaired t-test with Bonferroni-Dunn correction for multiple comparisons. Data are mean+/− SEM. *P < 0.05 **P < 0.01 ***<0.001. All *P* values are adjusted. For O-link analysis, the Benjamini, Krieger, and Yekutieli multiple nonparametric *t*-test was performed, ***adjusted *p* < 0.001, ****adjusted *p* < 0.0001.

Using comprehensive cellular immune phenotyping in patients with HPS-1, we investigated blood samples of 8 patients with genetically established HPS-1 and 12 controls using CyTOF (Table 1; Supplementary Table 1). Cell populations were resolved by unsupervised clustering of cell surface markers using FlowSOM into 13 metaclusters composed of 100 clusters (Fig. 1a; Supplementary Fig. 1)^17^. FlowSOM analysis identified a CD14^+^ meta-cluster that was significantly increased and a CD4^+^ meta-cluster that was significantly decreased in patients with HPS-1. Further analysis of the CD14^+^ metacluster identified 3 sub-clusters significantly associated with the HPS1 genotype (Fig. 1b). Two sub-clusters with markers indicative of classical CD14^+^ monocytes (cluster 11.1, cluster 11.2) were increased in patients with HPS-1 whereas a cell cluster with markers predictive of CD14^+^CD16^+^ intermediate monocytes (cluster 11.3) was decreased (Fig. 1b). The CD14^+^ classical monocyte clusters highly expressed CD64 and CD62L. In addition, we found a significant decrease in HPS-1 patients of a cell cluster with markers predictive of CD4^+^CD25^+^CD127^low^ T regulatory cells (Fig. 1b). To validate this observation, we manually gated this cluster (Supplementary Fig. 2a) and found that CCR6 expression is dampened in HPS-1 patient cells (Supplementary Fig. 2b) along with reduced CD45RA and CXCR4 (Supplementary Fig. 2c). The decrease in the median intensity of these proteins in HPS-1 may signify a dampened naïve cell pool (CD45RA^18^) and hampered migration (CXCR4^19^, CCR6^20^). A principal component analysis of these cell clusters further confirms a distinct immune cell phenotype that defines controls and HPS-1 patients (Fig. 1c).

**Table 1:** Patient population participating in this study.

Experiment	Group	N; (Male/Female)	Age (years ± SD)	Phenotype (% IBD;% lung fibrosis)
**CyTOF**	HPS-1	8 (4/4)	44 ± 12	25% PF
	Controls	12 (5/7)	47 ± 14	
**Serum proteomics**	HPS-1	11 (3/8)	39 ± 14	18% PF; 36% IBD
	Controls	50 (26/24)	35 ± 9	
**Transcriptomics**	HPS-1	4 (2/2)	36 ± 8	25% PF
	Controls	4 (2/2)	26 ± 1	
**Functional experiments**	HPS-1	10 (7/3)	41 ± 11	10% PF
	Controls	12 (8/4)	37 ± 12	
**Spatial transcriptomics**	HPS-1	1 (0/1)	8	IBD

*IBD* inflammatory bowel disease; *PF* pulmonary fibrosis.

Supervised clustering of monocyte populations confirms a significant increase of classical CD14^+^ monocytes in HPS-1 patients (Supplementary Fig. 3a, b). To further understand the phenotypic difference of CD14^+^ monocytes in patients with HPS-1 a viSNE analysis was performed (Fig. 1d). This demonstrated distinct populations of CD14^+^ monocyte populations in patients with HPS-1 compared to healthy controls. As observed in the FlowSOM analysis, CD14 + monocyte populations are defined by high expression of CD64 and CD62L in patients with HPS1 (Fig. 1d). The expression of CD64 in HPS-1 patient monocytes is 4.6-fold higher relative to healthy controls and the expression of CD62L is 1.9-fold higher relative to healthy controls (Fig. 1e). In summary, the CyTOF analysis suggests that HPS1 deficiency (in the absence of IBD) is associated with a distinct signature of inflammatory CD64^+^ CD62L^+^ monocytes and decreased regulatory T cells in the peripheral blood.

### Serum proteomics indicates a TNF/IL-1α inflammatory signature in HPS-1 patients

We next sought to understand relevant proteins and cytokines that are differentially represented in the serum of patients with HPS-1. We used an inflammation panel from O-link proteomics to interrogate 92 proteins in HPS-1 patient serum. Although there was heterogeneity among controls, HPS-1 patients clustered together in a hierarchical heatmap (Fig. 1f). A particularly enriched cluster of proteins in HPS-1 patient serum included the significantly increased TNF, IL-1α, CDCP1, IL-2, and TGF-α as well as heightened trends for IL-6 and IFN-γ (Fig. 1g; Supplementary Table 2)^21^. A pathway analysis of the proteins that cluster differentially between HPS-1 and controls highlights inflammatory pathways such as the TNF signaling cascade (Supplementary Table 3). A principal component analysis of the proteins confirms the segregation of HPS-1 patients from healthy controls but does not segregate HPS-1 patients with IBD, HPS-1 patients with PF or HPS-1 patients with neither of these complications (Fig. 1h). This analysis highlights an underlying cytokine dysregulation in HPS-1 patient serum, regardless of intestinal or pulmonary manifestations.

### Spatial transcriptomics unveils granuloma-associated signatures in HPS-1 patient tissue

Research into HPS intestinal tissue biology has been complicated by several factors. The disease is rare and the potential for excessive bleeding from thrombocyte defects in HPS-1 patients restricts research into tissue biology. We, therefore, leveraged formalin fixed paraffin embedded tissue archived from a colonic resection of a patient with granulomatous colitis. The HPS-1 patient was diagnosed at 2 years of age and had a segmental colectomy at 8 years due to refractory disease. Applying Nanostring GeoMx spatial transcriptomics, this material allowed us to study infiltrating immune populations as well as epithelial cells with distinct microanatomical regions. The size of the resection material allowed multiple intra-tissue comparisons between these microanatomical locations and cell types.

**Fig. 2:**
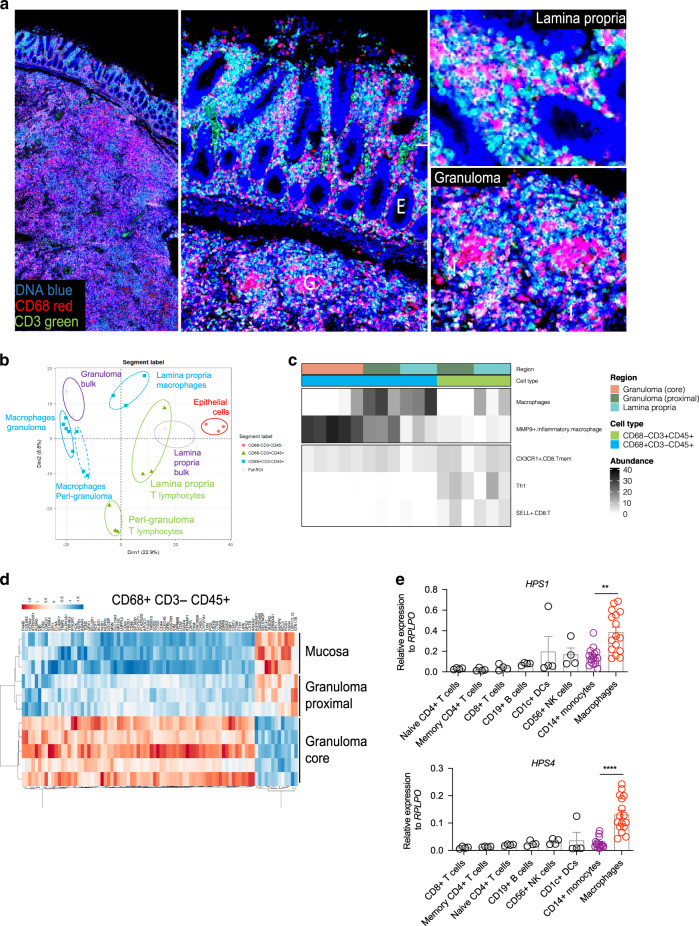
Spatial transcriptomics uncovers granuloma-associated signatures in the intestine of an HPS-1 patient. **a** HPS-1 gastrointestinal tissue used for spatial transcriptomics and immunofluorescently labelled for DNA, CD3, and CD68 staining. **b** Principal component analysis reveals cellular populations and anatomical regions as main drivers of variation. **c** Cell deconvolution of spatial transcriptomic profiles, mapped against a selection of cellular profiles provided by human adult gut scRNAseq atlas from the Human Cell Atlas project. **d** Heatmap of differentially expressed genes (*p* < 0.001) across the granuloma core, granuloma periphery and mucosa for CD68+CD3− CD45+cells. **e** *HPS1* and *HPS4* expression in different immune cell types (*n* = 4–16). Immune cells from peripheral blood were FACS sorted. Monocytes and monocyte-derived macrophages were isolated using CD14 beads and macrophage differentiation involved M-CSF treatment of monocytes for 5 days. Dot plot, mean and SEM are provided.**p < 0.01, ****p < 0.0001; Statistical significance was determined using a Tukey multiple comparisons test on log10-transformed data.

Staining cellular nuclei, CD45, CD3, and CD68 revealed submucosal granuloma formation and extensive infiltration of both submucosa and lamina propria with myeloid cells and T lymphocytes (Fig. 2a; Supplementary Fig. 4a, b). In principal component analysis, the location of the sampled areas accounts for the most variation in gene expression, demonstrating a distinct signature of the granuloma compared to lamina propria and peri-granuloma regions (Fig. 2b). In parallel, substantial variation was explained by the cellular difference between myeloid (CD68+) and non-myeloid (CD3+, CD45−) cells.

The identity of cells within the areas sampled in the HPS-1 patient was established by cell deconvolution against cell profiles derived from healthy and developing human gut^22^. This confirmed the specificity of our immunofluorescence-guided tissue sampling and it established a more pronounced inflammatory phenotype in the granuloma-associated macrophages when compared to lamina propria-associated macrophages (Fig. 2c). When compared to granuloma periphery or mucosa, CD68+ cells in the granulomata demonstrated increased gene expression in lysosome or macrophage-related genes such as CTSD, CD68, HEXB, ATP6V0A1, CTSK, LIPA, CD63 (Fig. 2d). Pathway analysis of the differentially expressed genes suggests not only plausible descriptions such as lysosome and innate immune system, but also lipid storage disorder and metabolic disease (Supplementary Fig. 4c). These results suggest a key role for monocytes and macrophages in HPS-1-associated inflammation.

### Multi-tissue single cell analysis suggests BLOC-3 pathway expression is high in myeloid cells

Gene expression in different tissues relevant for HPS pathology, i.e., the skin (albinism), PBMCs (monocyte activation and reduced regulatory T cells), the lung (fibrosis) and the gastrointestinal tract (inflammation) was examined.

As expected, melanocytes expressed all genes in the BLOC-3 pathway strongly (Supplementary Fig. 5a), and among PBMCs, the myeloid compartment and in particular CD14+ monocytes and CD1c+ dendritic cells exhibited high expression (Supplementary Fig. 5b). We validated these findings with a qPCR, showing that *HPS1* and *HPS4* are expressed in monocytes and especially upon differentiation into macrophages (Fig. 2e). Lastly, we investigated an idiopathic pulmonary fibrosis and a pooled colonic single cell dataset of patients with ulcerative colitis and non-inflamed controls. As for other tissues, there was primarily myeloid expression of the pathway genes in the lung and gut (Supplementary Fig. 5c, d).

Our multi-tissue gene expression analyses suggest that among immune cells, phagocytes strongly express the BLOC-3 machinery, consistent with our observations in HPS-1 patients featuring significant changes in monocyte number and phenotype.

### HPS-1 macrophages have a distinct immuno-metabolic signature

**Fig. 3:**
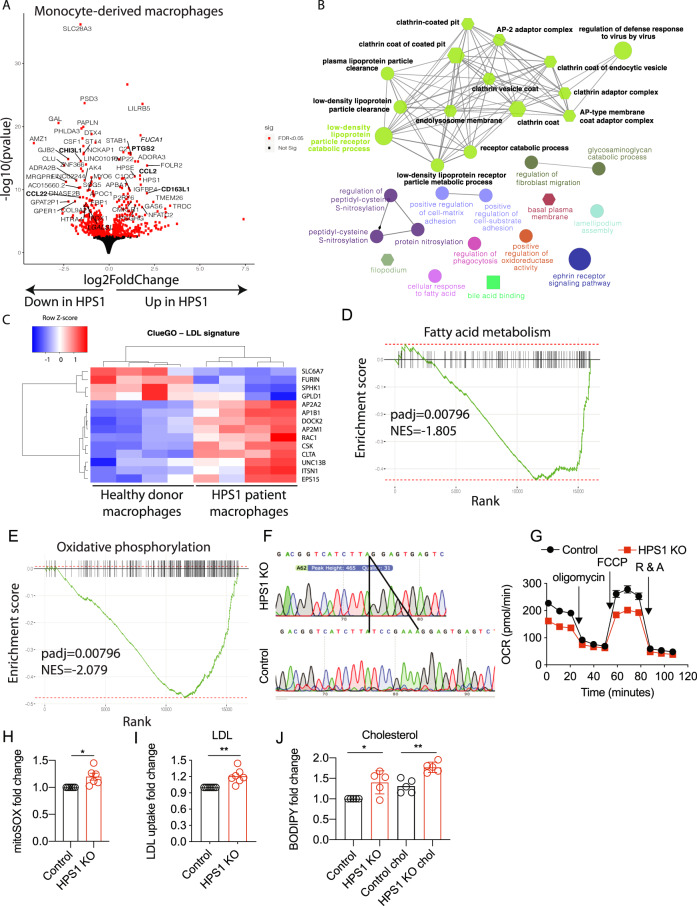
HPS1 deficiency leads to a dysregulated metabolic program. **a** Differential expression of HPS-1 and control macrophages. Known IBD-related cytokines are bolded. **B** Weighted Gene Correlation Network Analysis reveals a module highly associated with the HPS-1 disease trait; ClueGO was performed on the top 200 hub genes of this module. **c** Heatmap of the genes contributing to the LDL particle receptor catabolic signature. **d** Gene set enrichment analysis using hallmark pathways reveals a reduction in fatty acid metabolism. **e** Gene set enrichment analysis highlights decreased oxidative phosphorylation. **f** Sanger sequencing of HPS1 knockout HAP1 cells and control as a comparison. **g** Oxygen consumption rate of control and HPS1 knockout cells. Error bars in SEM. 12 replicates from one experiment, representative of two independent experiments. **h** MitoSOX staining on control and HPS1 knockout cells (*n* = 6). **i** Serum-starved HAP1 cells were treated with 2.5 μg/mL LDL-BODIPY for 3 h (*n* = 7). **j** HAP1 cells were stimulated with cholesterol for 2 h and stained with BODIPY 493/503 (*n* = 5). *p < 0.05, **p < 0.01, ****p < 0.0001; Ratio paired t-test on mean fluorescence intensity. Unpaired *t*-test on OCR log-transformed data. Padj = adjusted *p*-value; NES normalized enrichment score, R & A rotenone and antimycin A.

In order to investigate transcriptional signatures underlying HPS-1 disease pathology, we performed RNA-seq on primary monocyte-derived macrophages of control and patients with HPS-1 (Fig. 3a). In HPS-1 macrophages, 115 genes were upregulated and 106 downregulated with a significant adjusted *p*-value and a log2 fold change greater than 1 or smaller than −1, respectively (Supplementary Table 4). Weighted gene correlation network analysis identified a module highly correlated with HPS1 deficiency that is enriched in low density lipoprotein receptor (LDLR) catabolism (Fig. 3b, c). Within this network, LDL particle clearance, LDLR metabolism and clathrin coat terms appeared, showcasing the entire cycle of LDL metabolism as a prominent signature in HPS-1 macrophages. Pathway analysis for molecular function confirmed the LDL signature in HPS-1 macrophages (Supplementary Fig. 6a). Gene set enrichment and correlation network analysis established that gene expression indicative of fatty acid metabolism was downregulated in macrophages of patients with HPS-1 (Fig. 3d). Similarly, oxidative phosphorylation was dampened in HPS-1 macrophages, highlighting robust changes in metabolism (Fig. 3e).

To investigate whether the lipid metabolism signature is macrophage-specific we examined the transcriptional signature of organoid-derived lung epithelial cells with HPS1 deficiency (GSE121999). HPS1 knockout lung epithelial cells also show an enrichment of gene expression indicative of lipoprotein particle binding and a reduction in oxidative phosphorylation (Supplementary Fig. 6b, c). In order to functionally validate the role of altered immune metabolism in HPS1 deficiency, we generated an HPS1 CRISPR knockout in HAP1 cells, a myeloid derived haploid adherent cell line that expresses *HPS1* (Fig. 3f). Using HAP1 cells, we found that the oxygen consumption rate is reduced in HPS1 deficiency (Fig. 3g). Furthermore, HPS1 knockout cells had higher levels of mitochondrial reactive oxygen species (Fig. 3h).

In summary, these results suggest that the transcriptional control of LDL catabolism is present in both macrophages and epithelial cells and is altered in the disease state. Importantly, this defect in lipid metabolism is consistent with the spatial transcriptomic profile of granuloma-associated CD68 + cells. In parallel, we found that oxidative phosphorylation is dampened at the transcriptional and functional level in HPS1 deficiency.

### Aberrant metabolism in HPS1 deficiency

In agreement with altered lipid metabolism changes, low density lipoprotein (LDL) accumulation was significantly higher in HPS1-deficient HAP1 cells across multiple LDL stimulation time points (Fig. 3i; Supplementary Fig. 7a). Furthermore, HPS1 knockout cells accumulated significantly higher levels of cholesterol compared to controls, but phagocytosis rates were unchanged (Fig. 3j; Supplementary Fig. 7b).

**Fig. 4:**
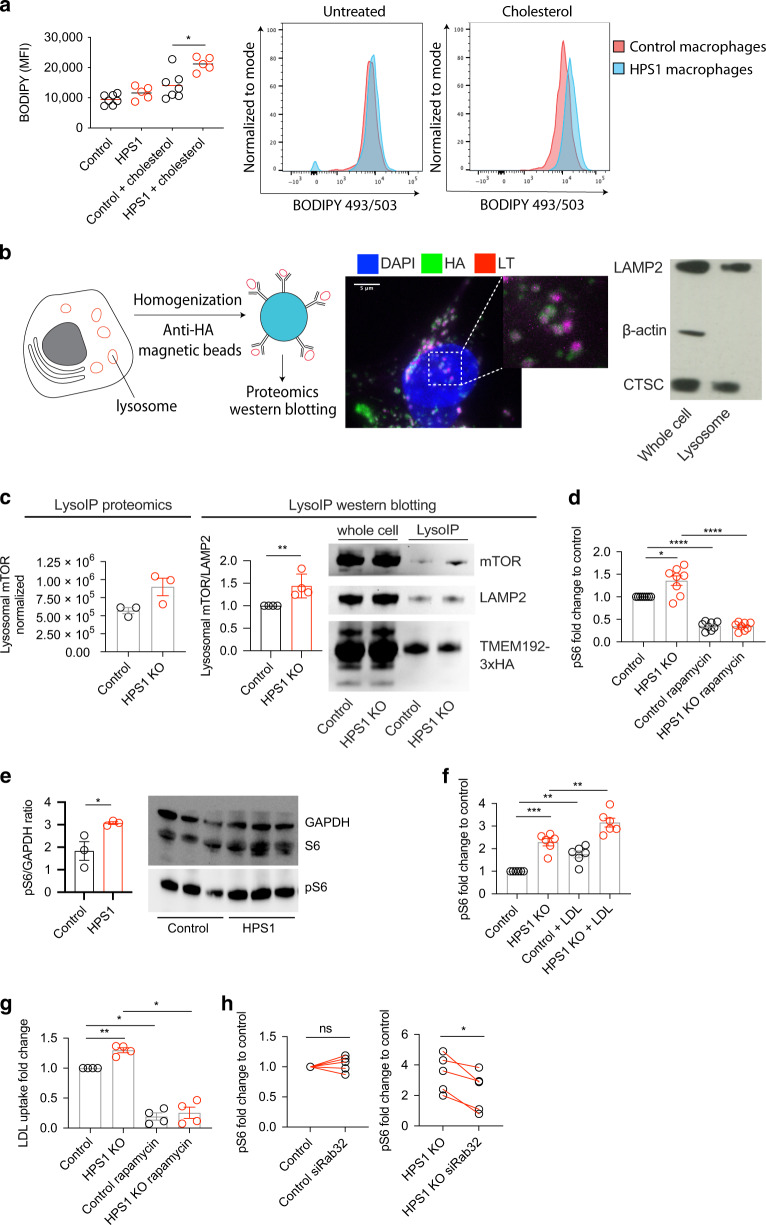
Rab32-mediated enhanced mTORC1 signaling in HPS1 deficiency. **a** Cholesterol stimulation was performed for 2 h prior to staining with BODIPY 493/503 in patient monocyte-derived macrophages (*n* = 5). Representative flow cytometry histogram plots. **b** Schematic of lysosomal immunoprecipitation and confocal microscopy of TMEM192-3xHA and LysoTracker (LT) in HAP1 cells. Lysosomal immunoprecipitation in cells approach was assessed through LAMP2 and CTSC Western blot. **c** LysoIP proteomics (*n* = 3) and Western blotting of lysosomal immunoprecipitation samples for mTOR, LAMP2 and TMEM192-3xHA tag (*n* = 4). **d** HAP1 cells were serum-starved overnight and treated with 50 μM rapamycin for 3 h prior to pS6 staining (*n* = 4). **e** Quantification of Western blot of control and HPS-1 patient macrophage samples for pS6 and GAPDH (*n* = 3 for healthy controls and HPS-1 patients). Quantification was performed using ImageJ. Western blot image used for quantification. **f** HAP1 cells were treated with 25 μg/mL LDL for 1 h and then stained for pS6 (*n* = 3). **g** LDL uptake in HAP1 cells using LDL-BODIPY for 1 h at 25 μg/mL were pre-treated with 50 μM rapamycin for 3 h (*n* = 4). **h** HAP1 cells were transfected with siRNA against Rab32 or a control pool and probed for S6 phosphorylation (*n* = 5). Statistics: Unpaired parametric test on log10-transformed mean fluorescence intensity data for **a**. Paired *t*-test on ratio data for **c**. Ratio paired *t*-test on mean fluorescence intensity (**d**, **f**, **g**). An unpaired *t*-test on ratio data was performed for the pS6/GAPDH quantification.

We next explored cholesterol metabolism in primary HPS-1 patient cells. As indicated by the neutral lipid BODIPY stain, exogenous cholesterol treatment in HPS-1 patient monocyte-derived macrophages resulted in a higher amount of lipids in HPS-1 patient cells (Fig. 4a). In summary, these data show that HPS1 deficiency affects LDL and fatty acid metabolism on a transcriptional level, leading to an accumulation of LDL and cholesterol.

### Rab32-mediated enhanced mTORC1 signaling in HPS1 deficiency

Since altered immuno-metabolism in HPS1 deficiency may be indicative of defective vesicle trafficking via the endo-lysosomal system, we studied the lysosomal compartment. We isolated lysosomes from control and HPS1 knockout HAP1 cells using magnetic immunoprecipitation, targeting the overexpressed TMEM192 tagged with HA^23^ (Fig. 4b). We stained cells for the HA-tag and LysoTracker to assess lysosomal co-localization of the tag with confocal microscopy, finding that the HA tag co-localized with lysosomes. The purity of intact lysosomes was confirmed by blotting for LAMP2, a lysosomal transmembrane protein, cathepsin C, a lysosomal lumen protein, and β-actin, which is excluded from the lysosome (Fig. 4b; Supplementary Table 5). Proteomic analysis of the lysosomal samples revealed enrichment of mammalian Target Of Rapamycin (mTOR) in the HPS1 knockout lysosomes as well as the associated LTOR1 and LTOR4 (Fig. 4c; Supplementary Fig. 7c). mTOR, a regulator of metabolism, growth and autophagy, senses lipids and amino acids and is activated at the lysosomal surface^24^. We validated the amplified mTOR abundance compared to LAMP2 in lysosomes of HPS1 knockout HAP1 cells (Fig. 4c). We observed significantly increased S6 phosphorylation, a surrogate marker of mTORC1 activation, which could be reversed using the mTORC1 inhibitor rapamycin (Fig. 4d). We confirmed these findings in HPS-1 patient macrophages, where pS6 levels were higher than healthy controls (Fig. 4e).

Enhanced LDL uptake reinforces the mTORC1 activation in HPS1 knockout cells (Fig. 4f). At the same time, rapamycin blunts LDL accumulation, suggesting that mTORC1 activation might further strengthen internalization and reduce lipid degradation (Fig. 4g). One major route of lipid uptake results from the transcription factor SREBP, which is controlled by mTORC1 and is sensitive to cholesterol levels^25^. SREBP target genes (*LDLR, FASN, HMGCR*) were either enhanced significantly or had an increased trend in HPS1 knockout cells (Supplementary Fig. 7d). However, the differences appear to be marginal and SREBP may not account for all lipid metabolism changes. In line with previous findings that augmented mTORC1 signaling controls cell size^26,27^, we found that HPS1 knockout HAP1 cells and HPS-1 patient macrophages are larger than controls (Supplementary Fig. 7e).

While Rab32 is a guanine nucleotide exchange factor target of BLOC-3^12^, it also helps anchor mTOR to the lysosome in a GTP-independent manner^28^. We hypothesize that HPS1 dictates the localization of Rab32 to different compartments and that in its absence, Rab32 preferentially helps attach mTOR to the lysosome. In fact, we found that Rab32 knockdown reverts the enhanced mTORC1 signaling in HPS1-deficient cells measured by pS6 (Fig. 4h; Supplementary Fig. 7f). These findings suggest that the heightened mTORC1 signaling in HPS-1 is mediated by Rab32. In summary, these data suggest a potentially pathogenic mechanism whereby HPS1 deficiency causes mTOR activation.

### HPS1 deficiency results in impaired cell intrinsic immunity

**Fig. 5:**
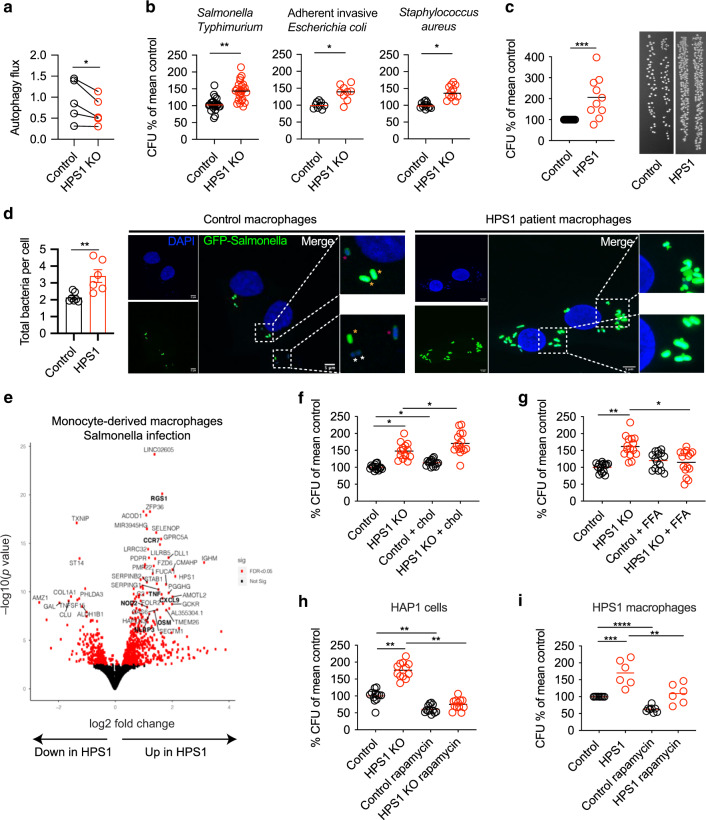
mTORC1 inhibition and fatty acids rescue HPS1 bacterial clearance defects. **a** HAP1 cells were treated with 10 nM bafilomycin A1 for 2 h and the FlowCellect autophagy kit was used (*n* = 5). **b** The gentamicin protection assay was performed in HAP1 cells using GFP-*Salmonella* Typhimurium (*n* = 8), Adherent Invasive *E. coli* (*n* = 3) or *Staphylococcus aureus* (*n* = 4). **c** Gentamicin protection assay of healthy donor and HPS-1 patient-derived macrophages (*n* = 10). Representative agar plate of gentamicin protection assay. **d** Quantification of number of bacteria in each cell using confocal microscopy in control and HPS-1 macrophages (7 controls, 6 HPS-1 patients). The average bacteria per cell in each donor was used for statistical tests, where we performed an unpaired t-test. Representative confocal images; scale bar 5 μm. White asterisk denotes DAPI + GFP- bacteria; yellow asterisk DAPI + GFPbright bacteria; purple asterisk DAPI + GFPdim bacteria. **e** Differential expression of HPS1 patient and control macrophages following *Salmonella* Typhimurium infection (*n* = 4). **f** HAP1 cells were stimulated with 50 μM cholesterol for 2 h prior to the gentamicin protection assay (*n* = 5). **g** HAP1 cells were treated with free fatty acids (FFA) for 2 h prior to a gentamicin protection assay (*n* = 5). **h** HAP1 cells were treated with 50 μM rapamycin for 3 h followed by the gentamicin protection assay (*n* = 4, *Salmonella* Typhimurium). **i** Macrophages were treated with 50 μM rapamycin for 2 h prior to *Salmonella* Typhimurium infection and the gentamicin protection assay (*n* = 6); each dot represents one patient. Significance was determined using a paired t-test on the autophagy flux values. For gentamicin protection assays, we used a ratio paired t-test for HAP1 cells and an unpaired t-test on log-transformed data for patient macrophages. *p < 0.05, **p < 0.01, ***p < 0.001, ****p < 0.0001.

Since mTORC1 is a key regulator of anti-microbial autophagy and lysosomal degradation, we investigated the impact of HPS1 deficiency on autophagy flux. HPS1-deficient cells demonstrated lower autophagy flux compared to controls (Fig. 5a). In parallel, HPS1 knockout HAP1 cells exhibited reduced anti-microbial activity against *Salmonella enterica* serovar Typhimurium, Adherent Invasive *E. coli* and *Staphylococcus aureus* in comparison to the control (Fig. 5b). Importantly, HPS-1 patient macrophages also showed a significantly impaired ability to eliminate bacteria (Fig. 5c). This finding was confirmed by confocal microscopy showing significantly higher numbers of *Salmonella* Typhimurium in HPS-1 patient macrophages (Fig. 5d). In addition, more GFP + bacteria were present in HPS-1 patient macrophages, but the number of bacteria in the process of being degraded (GFPdim) or that were already degraded (DAPI + GFP-) were unchanged (Supplementary Fig. 8a). At the same time, lysosomal function is intact in HPS1 deficient cells, measured through DQ-ovalbumin (Supplementary Fig. 8b). While bacterial handling is hampered in HPS1 knockout cells, phagocytosis was not altered, highlighting the impact of HPS1 deficiency on bacterial elimination intracellularly. These results highlight that intrinsic lysosomal function is intact, but bacteria can evade bacterial clearance and replicate more easily in HPS1 deficiency.

Given that response to bacteria is crucial for tissue homeostasis at mucosal surfaces, we used RNA-seq to uncover transcriptional signatures associated with defective bacterial clearance in HPS1 patient macrophages (Fig. 5e). We identified enhanced expression of pro-inflammatory mediators, such *OSM*, *TNF, CCR7, NOD2, NLRP3 and RGS1*, after *Salmonella enterica* serovar Typhimurium infection. Additionally, MHC binding and cytokine activity were enhanced pathways in HPS1 patient macrophages following infection, while oxidative phosphorylation remained dampened (Supplementary Fig. 8c, d). In combination with the serum proteomics data, this highlights a significant inflammatory signature in HPS-1 patients, which may in part be secondary to defects in bacterial handling.

### mTORC1 inhibition and fatty acids rescue defective anti-microbial activity in HPS1 deficiency

HPS1 deficiency results in altered immuno-metabolism and amplified mTORC1 signaling, which may cause defective anti-bacterial activity. We found that cholesterol, which accumulates in multiple lysosomal storage disorders, leads to reduced bacterial clearance in both control and HPS1 knockout cells (Fig. 5f). Since lipid droplets (LD) release free fatty acids upon breakdown, we treated the HPS1 knockout cells with free fatty acids to investigate whether defective LD breakdown contributes to hampered bacterial clearance. We observed the reversion of the bacterial clearance defect in HPS1 deficiency by free fatty acid supplementation, which may be mediated through mTORC1 (Fig. 5g; Supplementary Fig. 8e). In order to investigate the role of fatty acid oxidation in HPS-1, we treated HPS1-deficient and control cells with etomoxir, a CPT1 inhibitor^29^. Etomoxir-treated control HAP1 cells had a higher bacterial load compared to control cells, highlighting the importance of fatty acid oxidation in bacterial clearance (Supplementary Fig. 8f). However, etomoxir did not have an effect on HPS1-deficient cells, since fatty acid oxidation may already be defective. These results indicate that fatty acid availability is required for bacterial handling. We found that RAB32 depletion results in an anti-microbial cellular defect at baseline (Supplementary Fig. 8g). However, this depletion does not further exacerbate the microbial elimination defect in HPS1 knockout cells, since the BLOC-3/Rab32 antimicrobial pathway is already impaired. These results highlight the instrumental role of metabolism in host defense, and that this balancing act is dysregulated in HPS1 deficiency.

Since Rab32 facilitates mTORC1 signaling in HPS1 deficiency, we wondered whether mTORC1 inhibition could rescue the bacterial clearance defect. Indeed, treatment of HPS1 knockout HAP1 cells with the mTORC1 inhibitor rapamycin restored anti-microbial activity (Fig. 5h). We validated this finding in HPS-1 patient monocyte-derived macrophages and showed that rapamycin also rescued the bacterial handling defect in patient cells (Fig. 5i). These findings suggest that altered metabolism and enhanced mTOR signaling are central to the cellular defects in HPS1 deficiency and that rapalogues may act as a therapeutic avenue for HPS-1 patients.

## Discussion

**Fig. 6:**
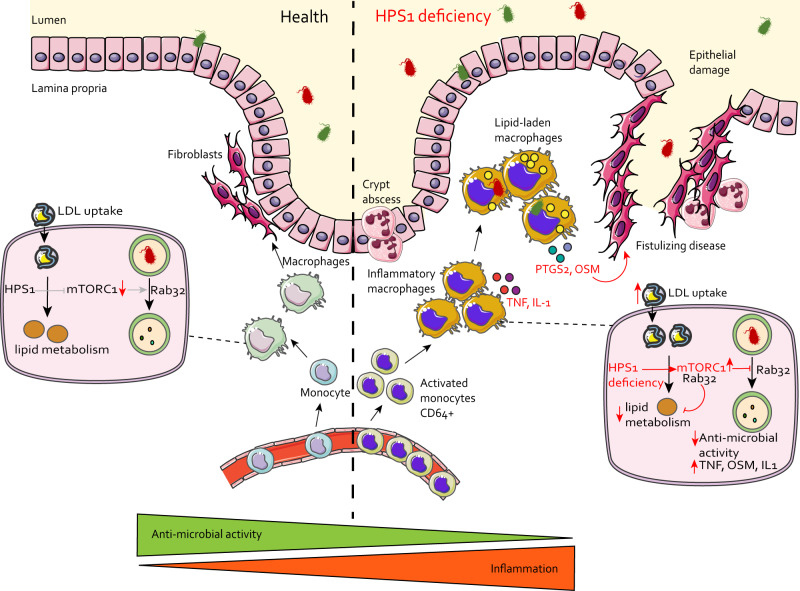
Summary of intestinal inflammation and molecular mechanisms in HPS1 deficiency. In health, monocytes from the blood differentiate into macrophages, which interact with regulatory T cells and fibroblasts, promoting homeostasis. On a molecular level, lipid droplet metabolism and lower mTORC1 levels facilitate the degradation of microbes, where Rab32 plays a key role in phagosome maturation. In HPS1 deficiency, activated monocytes are recruited from the blood, differentiating into macrophages that secrete inflammatory mediators such as TNF, IL-1, OSM and PTGS2. On an intracellular level, high levels of mTORC1 activity mediated through Rab32 and deregulated immune metabolism dampen anti-microbial activity. The suppressed degradation of bacteria and increased cytokine production in HPS-1 patients can lead to tissue inflammation involving crypt abscesses, epithelial barrier damage, and fistulizing disease. Figure elements are derived from Servier Medical Art^80^.

In this study, we perform a multi-omic analysis to define the cellular underpinnings that cause inflammation and defective anti-microbial activity in patients with HPS1 deficiency (Fig. 6). We found the presence of inflammatory monocytes and macrophages in HPS-1 patients and that myeloid cells have the highest expression of the BLOC-3 pathway across tissues involved in HPS pathology. HPS-1 patient macrophages have impaired bacterial clearance, originating from alterations in immunometabolism and mTOR signaling, which is involved in metabolic sensing. Importantly, we show that inhibiting mTOR as a metabolic checkpoint can override the genetic deficiency and restore anti-microbial activity. Correcting this metabolic regulator has potential therapeutic implications.

We provide comprehensive immune phenotyping of HPS-1, identifying an expanded inflammatory CD64^+^ CD62L^+^ monocyte compartment in HPS-1 patient peripheral blood. CD64 and CD62L have been described as markers of inflammatory circulating monocytes in several diseases and infections, including COVID-19 and rheumatoid arthritis^30–32^. An increase in inflammatory cytokines such as TNF and IL-1α in HPS-1 patient serum and *TNF* and *OSM* in infected HPS-1 patient macrophages are relevant findings since these cytokines are markers of severe tissue inflammation in the intestine^33,34^. Furthermore, our case study of an HPS-1 patient tissue resection highlights the presence of inflammatory and potentially dysfunctional macrophages at the site of injury (granulomata are not present in healthy controls). The study illustrates the potential of spatial transcriptomics to differentiate mRNA expression profiles of immune cells at different microanatomic niches. Our study extends the findings of increased cytokines such as TNF and IFN-γ in HPS pulmonary fibrosis serum, highlighting a cluster of proteins significantly increased in HPS-1 patient serum^14^.

Our data add to the array of genetic and functional studies (*NOD2, LRRK2, IRGM, ATG16L1*) that highlight host-pathogen interactions as key determinants of gut homeostasis by unveiling the mechanism underlying immune dysregulation in HPS-1^35–40^. We identify changes in cholesterol and fatty acid metabolism in HPS1 deficiency as well as enhanced mTORC1 signaling, which is mediated through Rab32. Interestingly, Hps1-deficient mice accumulate cholesterol and have more lipid droplets in liver cells, showcasing the effects of this mechanism in vivo^41^. In addition, Rab32 is a lipid droplet resident protein and has demonstrated roles in lipid metabolism, such as controlling lipid droplet size^42–44^. With these studies in mind, we found that free fatty acids, which might not be released from lipid droplets efficiently in HPS1 deficiency, could stimulate bacterial degradation in HPS1 knockout HAP1 cells. In addition, we found that HPS1 deficiency resulted in diminished oxidative phosphorylation and heightened mitochondrial ROS production compared to controls, drawing parallels with observations in IL-10 signaling defects^45^.

Previous studies have suggested that Rab32 deficiency hampers microbial clearance and that Rab32 delivers itaconate to phagosomes^16,46,47^. At the same time, Rab32 helps anchor mTOR to the lysosomal surface, regardless of GTP/GDP status^28^. Since BLOC-3 acts as a guanine nucleotide exchange factor for Rab32^12^, we would expect that HPS1 deficiency would result in an accumulation of Rab32-GDP, which could stimulate mTORC1 signaling. Intriguingly, BLOC-3 overexpression increases Rab32 localization to *Salmonella*-containing vacuoles, lending credence to the hypothesis that BLOC-3 controls Rab32 localization for bacterial clearance^48^. While HPS1 and HPS4 can be recovered in phagosomes, Rab32 localizes to both phagosomes and lipid droplets^44,49^. It appears plausible that BLOC-3, through aiding the exchange of GDP for GTP, dictates not only the localization, but also the function of Rab32, driving mTORC1 activation in BLOC-3 deficiency. Concurrently, the GTPase-activating protein RUTBC1 can inactivate Rab32, adding another regulatory layer in the BLOC-3 pathway^50^. This central circuit results in aberrant bacterial clearance and a dysregulated cytokine response. We note that both the lung and intestine are constantly exposed to external pathogens as well as resident microbiota. We believe that altered handling of bacteria by tissue-resident myeloid cells may induce tissue-specific interactions relevant for the intestinal inflammation and pulmonary fibrosis observed in HPS-1 patients^33,51^. Intriguingly, human bronchial epithelial cells are activated in response to the bacterial component flagellin through mTOR, resulting in the expression of inflammatory mediators^52^. Therefore, mTOR may act as an anti-microbial checkpoint and its modulation presents an attractive therapeutic target for mucosal sites where host-microbe interactions dominate.

Several genetic conditions highlight the role of a metabolic checkpoint in intestinal inflammation. In a setting of IL-10 and IL-10R deficiencies, where patients present with infantile-onset IBD, macrophages develop deregulated glycolysis associated with increased mTOR activation and aberrant inflammasome activation^45^. On the other hand, anti-microbial activity, inflammasome activation and fatty acid oxidation are interlinked in LACC1 deficiency, which is implicated in Crohn’s disease^53^. Defects in LACC1 caused hampered autophagy in macrophages and impaired mitochondrial respiration^54^. In this context, our experiments provide novel insights by highlighting the role of HPS1 and RAB32 as a rheostat for the mTOR system. Patients with Niemann-Pick Type C1 (NPC1) develop Crohn’s-like intestinal inflammation and have a microbe elimination defect^55^. NPC1 deficiency causes aberrant cholesterol accumulation associated with increased mTORC1 signaling^56–58^. Interestingly, lysosomal acid lipase deficiency causes a cholesteryl ester storage disease but not Crohn’s disease^59^. As such, we would predict that the accumulation of cholesteryl esters, unlike cholesterol, does not heighten mTOR signaling, despite the presence of lipid droplets in macrophages, and may explain the shared phenotype across these genetic conditions.

A recent study suggested that Hps1-deficient murine Paneth cells are unable to secrete lysozyme following LPS stimulation^60^, showcasing an additional factor that may contribute to inflammation. Multiple questions remain to be answered to gain full insight into HPS-1 pathology. For instance, identifying translocating microbes into the lamina propria can pinpoint microbial triggers and increase our understanding of the penetrance of IBD in these patients. A reproducible murine Hps1 colitis model could facilitate these investigations and the validation of novel therapies for HPS-IBD, including rapamycin.

We show that deranged metabolism, Rab32, and mTORC1 activation underlie HPS1 deficiency. As a consequence of a defective metabolic checkpoint, HPS-1 patient-derived macrophages are unable to degrade microbes. Lipid-laden macrophages and granulomata are histological illustrations of these defective processes in vivo. Our findings provide evidence for a therapeutically targetable BLOC-3-Rab32-mTOR circuit that affects anti-microbial activity. These findings showcase that granuloma formation and lipid metabolism changes observed in IBD can be disentangled through understanding rare diseases such as Hermansky-Pudlak Syndrome.

## Methods

### Patient population and controls used in this study

The study was designed to identify the immune phenotype of HPS1 deficiency and to pinpoint cellular dysregulations that may contribute to disease pathology. All sample collection was approved by the institutional review boards (Oxford IBD cohort study and a sub-project to investigate rare diseases; 11/YH/0020, 16/YH/0247, IRB 11-01669 study protocol was used for collection of samples at Mt Sinai, NY; IRB study protocol 1250116 from the University of Puerto Rico was used for the collection of tissue). Written informed consent was provided by all patients or guardians. Only anonymous patient information was processed and since this is a rare disease, we used all HPS-1 samples that were available, as outlined in Table 1. Patients had clinical phenotypes consistent with HPS-1 and previously confirmed through genetic testing at a commercial lab or the NIH. Control PBMCs were obtained from healthy volunteers via the Oxford GI biobank.

### HAP1 cell culture

HAP1 cells (Horizon Discoveries) were maintained in DMEM (Sigma) with 10% fetal bovine serum (Sigma) at 37 °C and 5% CO_2_. Cells were detached with 0.05% Trypsin-EDTA (Gibco). Cells were regularly checked for mycoplasma.

### PBMC isolation and macrophage differentiation

Healthy donor blood samples were obtained as part of the IBD cohort (09/H1204/30) and GI biobank (16/YH/0247) and HPS patient blood from Mount Sinai, NY. Lymphoprep (Axis-Shield) was used to perform density gradient centrifugation. Monocytes were enriched from PBMCs using the adherence method^55,61^ and treated with 100 ng/mL M-CSF (R&D systems) in RPMI-1640 (Sigma), 10% FCS (Sigma) and 1% Penicillin/Streptomycin (Sigma) for 5 days to differentiate into macrophages.

### CRISPR/Cas9 gene editing and siRNA transfections

gRNA sequences were obtained from previously reported guides^62^. RNP complexes were generated according to the manufacturer’s protocol using HiFi Cas9 (IDT) and transfections were performed using lipofectamine 2000 (ThermoFisher).

For siRNA transfections, we used the Rab32 siGENOME (Horizon Discovery, SMARTPool, M-009920-02-0005) and the non-targeting siRNA pool #1 (Horizon Discovery, D-001206-13-05). Cells were transfected with 40 nM siRNA and INTERFERin (Polyplus transfection, 409-10) according to the manufacturer’s protocol. Experiments were performed 72 h following transfection.

### Lentiviral production and transduction

Plasmids used for lentiviral transduction were pLJC5-Tmem192-3xHA (a gift from David Sabatini, Addgene plasmid 102930), psPAX2 (a gift from Didier Trono, Addgene plasmid 12260) and pMD2.G (a gift from Didier Trono, Addgene plasmid 12259). We transfected HEK293T cells (ATCC) at 60–80% confluence using lipofectamine (ThermoFisher) and Opti-MEM (Gibco). Viral supernatant was collected after 48 and 72 h, filtered through a 0.45 μm filter, and used to transduce HAP1 cells with 6 μg/mL polybrene (TR-1003-G, Sigma). After 48 h, cells were positively selected with 0.5 μg/mL puromycin (P8833, Sigma).

### RNA extraction, cDNA synthesis, and qPCR

RNA extraction was performed using RNeasy mini kit (Qiagen), cDNA synthesis was performed using 1 μg RNA and High Capacity cDNA Reverse Transcription Kit (Applied Biosystems). The Taqman probes (Life Technologies) used in this study include: *Rab32* (Hs00199149_m1), *RPLPO* (Hs00420895_gH), *HPS1* (Hs00945781_g1), *HPS4* (Hs01031019_m1), *LDLR* (Hs01092524_m1), *FASN* (Hs01005622_m1), *HMGCR* (Hs00168352_m1). Expression was determined by the ΔCt method and normalized to *RPLPO* expression levels.

### Oxygen consumption rate

Oxygen consumption rate (OCR) of the HAP1 cells was quantified on a XF96 extracellular flux analyzer (Seahorse Bioscience) using the Seahorse XF Cell Mito Stress kit (Agilent, catalog number 103015-100) and according to the manufacturer’s protocol. The Seahorse XFe96 FluxPak (Agilent, catalog number 102601-100) and 50,000 HAP1 cells were plated in Seahorse base media (Seahorse XF base medium (Agilent, catalog number 102353-100) in 12 replicates per condition with 1% FCS, 1 mM glutamine (Sigma, catalog number 59202C-100ML) and 2 mM sodium pyruvate (Sigma, catalog number S8636-100ML). Plates were incubated in a CO_2_-free incubator at 37 °C for 1 h prior to acquisition on the Seahorse machine. Basal respiration (prior to oligomycin addition) and maximal respiration (following FCCP treatment) were calculated by subtracting non-mitochondrial oxygen consumption (values after rotenone & antimycin A addition).

### Western blotting

Cells were lysed in pH 7.5 buffer containing 50 mM Tris, 150 mM NaCl, 2 mM EDTA, 50 mM NaF, 1% Nonidet-P40 and 2 mM Na4P2O7 plus protease inhibitors (Roche). Cell lysates were loaded on NuPAGE® Novex® 4–12% Bis-Tris Protein Gels, 1.0 mm, 10 well (NP0321BOX) and running buffer (Life Technologies) using standard protocols. Blotting was done on a PVDF membrane (Invitrolon, LC2005 Thermo Fisher) using transfer buffer (Life Technologies). The following antibodies were used: anti-LAMP2 (clone H4B4, Santa Cruz Biotechnology), anti-HA (clone C29F4, Cell Signaling), anti-CTSC (clone D-6, Santa Cruz Biotechnology), anti-mTOR (2972, polyclonal, Cell Signaling), anti-pS6 Ser235/236 (polyclonal, Cell Signaling, 2211S), anti- GAPDH (clone 14C10, HRP conjugate, Cell Signaling, 3683S) and anti-β-actin (8H10D10, HRP conjugate, Cell Signaling). Signals were detected using HRP conjugated secondary antibodies (Cell Signaling) followed by enhanced chemiluminescence (ECL, GE Healthcare Life Science) and were recorded on a Biorad Chemidoc imaging system.

### Confocal microscopy

100,000 macrophages were seeded on tissue culture slides (Sarstedt) and infected with GFP-Salmonella, MOI 1:20, for 1 h followed by 100 μg/mL gentamicin treatment for 1 h. Cells were fixed for 10 min at 37 °C in 4% paraformaldehyde (ThermoFisher). Similarly, 80,000 HAP1 cells were stained with LysoTracker (ThermoFisher, L12492, 1:2000) for 1 h at 37 °C. Cells were stained with an anti-HA AF488 antibody (ThermoFisher, clone 16B12, A21287) for 1 h following fixation and permeabilization with 0.01% Triton for 10 min at room temperature. Cells were counterstained with DAPI and sealed with vecta shield (Vector labs). Images were acquired on a Zeiss LSM 880 microscope using a 63X/1.4 oil objective lens.

### Lysosomal immunoprecipitation

Lysosomal immunoprecipitation was performed as described previously with minor modifications^23^. Lentivirus-transduced HAP1 cells were rinsed with PBS and scraped in KPBS. A cell sample was kept aside as a control while the rest was homogenized with 25 strokes of the dounce glass tissue homogenizer (VWR, #71000-516) on ice. Homogenate was centrifuged at 600 g 2 min 4 °C and the supernatant was transferred to a tube containing 50 μL Pierce anti-HA magnetic beads (ThermoFisher, #88836), mixed and put onto a gentle rotator for 5 min at 4 °C. Tubes were placed onto a magnet, washed in KPBS 3 times and dry pellets of beads-lysosomes were frozen in −80 °C for proteomic analysis. A small volume of sample was taken during the last KPBS wash for Western blot analysis.

### Gentamicin protection assay

The gentamicin protection assay was performed as described previously^55,61^. Prior to infection, macrophages or HAP1 cells were treated with 50 μM rapamycin (Cayman Chemical), lipid mixture 1 as free fatty acids (Sigma, L0288), 50 μM cholesterol (Sigma, C8667) for 2.5 h or 50 μM etomoxir overnight (Sigma, E1905). HAP1 cells were infected at 1:100 MOI and macrophages at 1:10 MOI for 1 h with GFP-*Salmonella* Typhimurium (NCTC 12023), Adherent Invasive *E. coli* strain LF82 or *Staphylococcus aureus* (NCTC 657) was performed. Cells were then treated with 100 μg/mL gentamicin (Sigma) for 2 h and subsequently lysed with 1% Triton X-100 (Sigma). Lysates were then plated using the track method on LB agar plates. CFU were counted on the following day.

### Flow cytometry

#### Probes and dyes

For LDL internalization, cells were serum-starved overnight and stimulated with 2.5 μg/mL or 25 μg/mL LDL-BODIPY (L3483, ThermoFisher) or unlabeled LDL (L3486, ThermoFisher) for indicated time points. For phagocytosis measurements, we treated cells with pHrodo Red *E. coli* Bioparticles (P35361, ThermoFisher) for indicated time points according to the manufacturer’s protocol. For cholesterol stimulation and BODIPY staining, macrophages were treated with 5 X cholesterol or 50 μM cholesterol (Cholesterol, C8667, Sigma; Cholesterol lipid concentrate 250 X, 12531018, ThermoFisher) for 2 h and stained with 1 μg/mL BODIPY 493/503 (D3922, ThermoFisher) for 20 min at 37 °C. Lysosomal function was monitored using DQ Ovalbumin (D12053, ThermoFisher) for 2 h following 25 μg/mL LDL stimulation for 1 h. In order to measure mitochondrial ROS, we used the MitoSOX Red mitochondrial superoxide indicator (Thermo Fisher, M36008) according to the manufacturer’s instructions. Specifically, cells were treated with 5 μM MitoSOX Red in HBSS for 15 min at 37 °C.

#### Autophagy flux

Autophagy was measured using the FlowCellect Autophagy LC3 Antibody-based Assay Kit (Millipore, FCCH100171) and according to the manufacturer’s instructions. Cells were treated with 10 nM bafilomycin A1 (Merck, catalog number SML1661) for 2 h. Cells were then washed in Hanks’ Balanced Salt Solution (HBSS), detached, and washed in 1 X assay buffer. Cells were resuspended in 1 X reagent B solution to permeabilize cells and spun down immediately. Kit-based 1 X LC3-FITC staining was performed at room temperature for 30 min. Autophagy flux was calculated as: (bafilomycin A1 – untreated) / untreated.

#### Phosflow

Cells were treated with 50 μM rapamycin (Cayman Chemical) for 3 h. For phosflow, Fixable Viability Dye eFluor 780 (65-0865-14, eBioscience) was used prior to fixing with Cytofix (554655, BD Biosciences). Cells were permeabilized with perm buffer III (558050, BD Biosciences) and stained with pS6 pS235/pS236 (AF488, clone N7-548, BD Biosciences, 1:10 dilution).

#### Immune cell sorting

The sorted immune cell cDNA was used from a previous study in the lab^61^.

### Lysosomal proteomics

Proteins phosphorylated in vitro were subjected to SMART Trypsin digest (Thermo Fisher) treatment according to the manufacturer’s instructions, desalted using SepPak reversed-phase columns (Waters), and injected into an LC-MS/MS platform (Dionex Ultimate 3000 nano LC and Q-Exactive). Sample separation was undertaken using a 50-cm-long EasySpray column (ES803; Thermo Fisher) with a 75-μm inner diameter and a gradient of 2 to 35% acetonitrile in 0.1% formic acid and 5% DMSO with a 250 nL/min flow rate for 60 min. MS1 spectra with a resolution of 70,000 and an ion target of 3e6 were acquired for a maximum of 100 ms. MS/MS data were acquired after isolation with a mass window of 1.6Th and fragmentation at 28% normalized collision energy (HCD, Resolution 17,500). PEAKS V.8.5 (Bioinformatics Solutions) and a Uniprot/Trembl database (UP000005640 [*homo sapiens*] and UP000054420 [*salmonella enterica*]) were used to analyze the LC-MS/MS data set to identify phosphorylation (S, T, Y), as well as oxidation (M) and deamidation (N, Q). Mass tolerance was 10 ppm for precursor and 0.05 Da for fragment mass, 3 missed cleavages with a peptide level false discovery rate set to 1%. Freestyle 1.3 (Thermo Fisher) was used to generate extracted ion chromatograms of relevant peptides that were quantified after Gaussian smoothing (five data points). Progenesis QI (Waters) based label-free quantitation results were imported into Perseus 1.5.2.4^63^. Quantitative data was log2 transformed and normalized by median subtraction and missing values were imputed based on normal distribution. The proteomics data have been deposited to the ProteomeXchange Consortium via the PRIDE partner repository with the dataset identifier PXD024435 and 10.6019/PXD024435^64^.

### O-link proteomics

The O-link assay was performed according to the protocol of the O-link INFLAMMATION (#11-00866) panel, which consists of 92 paired oligonucleotide antibody-labeled probes. 1 μl of patient plasma was mixed with 3 μl O-link incubation mix and incubated at 4 °C overnight. The next day the O-link extension reagent mix was added to each well, vortexed, spun down and placed into a thermal cycler for pre-amplification over 1.5 h. 2.8 μl from each well is then mixed with 7.2 μl of detection mix and placed on a 96-96 Dynamic Array Integrated Fluidic Circuit chip along with the oligonucleotide pairs. The chip is processed through the Fluidigm BioMark qPCR reader per the supplier’s protocol (https://www.olink.com). Sample data quality is normalized using O-link’s Normalized Protein eXpression Manager software. We performed a multiple unpaired t-test and proteins with a *p* < 0.05 were considered significant. For the downregulated proteins in HPS1 serum we used gProfiler to identify relevant pathways (https://biit.cs.ut.ee/gprofiler/gost).

### CyTOF processing and analysis

Prior to CyTOF assays previously optimized antibody mixtures were prepared in cell staining media (CSM, Fluidigm). Antibody lists are outlined in Supplementary Table 1. Whole blood was collected from patients into a sodium heparin vacutainer and processed within one hour. 1.2 ml of blood was mixed with 3 ml of Thaw-Lyse buffer (Smart Tube Inc.) and incubated for 10 min at room temperature. Samples were centrifuged and resuspended in 10 ml Thaw-Lyse buffer and incubated for 10 min at room temperature. Samples were again centrifuged and washed in CSM. Each sample was then resuspended in 800 μl of 1X Barcode Perm Buffer (Fluidigm Inc.). Compatible Pd-barcodes were thawed, resuspended in 100 μL of 1X Barcode Perm Buffer and added to the samples, followed by 30 min on ice and washing in CSM and pooled together. Each barcoded set of samples was resuspended in 100 μL CSM containing 100 U ml^−1^ heparin (Sigma) to block non-specific MaxPar Antibody binding. A titrated surface antibody panel (Supplementary Table 1) designed to allow identification of all major immune subsets was prepared in 100 μl CSM, filtered through a 0.1 μm spin filter (Amicon) and added to the sample. Samples were stained for 30 min on ice, washed with CSM and fixed with 2% formaldehyde (Electron Microscopy Sciences) in PBS. The samples were washed and permeabilized with 1 ml ice-cold 100% methanol. Samples were incubated on ice for 30 min (or transferred to −80 °C for long-term storage), washed twice with CSM, and resuspended in 100 ul CSM with heparin. Samples were washed with CSM and incubated for 30 min in 2% formaldehyde in PBS containing 0.125 nM Ir nucleic acid intercalator (Fluidigm). The samples were washed and stored as pellets in CSM until CyTOF acquisition.

Immediately prior to acquisition, samples were washed with PBS and deionized water, counted and resuspended at a concentration of 1 million cells ml^−1^ in water containing a 1/20 dilution of EQ. 4 Element Beads (Fluidigm). Samples were acquired on a CyTOF2 Mass Cytometer equipped with a SuperSample fluidics system (Victorian Airships) to facilitate bulk sample acquisitions. Samples were acquired at a flow rate of 0.045 mL min^−1^ and an event rate of <400 events per second. CyTOF FCS files were concatenated and normalized using the bead-based normalization tool in the Helios software (Fluidigm), the barcoded samples were automatically deconvoluted and cross-sample doublets were filtered using a Matlab-based debarcoding tool^65^ and the resulting files were uploaded to Cytobank. Cell events were identified as Ir191/193 positive events, and residual Ce140+ normalization beads were excluded.

All analyses of CyTOF samples were performed using Cytobank software. High-dimensional analysis used to map the multi-dimensional data into 2-dimensional space include a self-organizing map (SOM, FlowSOM) and t-distributed stochastic neighbor embedding (viSNE). All high-dimensional analyses were performed on singlets and the data files analyzed together to ensure cells clusters are stable across experiments. In all analyses cells were clustered using surface antibody markers. For viSNE and FlowSOM analysis all samples were set to equal sampling at 30,000 events per sample and run per default parameters for the software. For FlowSOM metacluster number was optimized to minimize metaclusters composed of a single cluster. Major immune populations were identified based on canonical marker expression patterns. Principal component analysis of FlowSOM metaclusters was performed using SPSSv26 based on eigenvalue > 1 without rotation. A heatmap of surface marker expression was generated using the heatmap.2 package in R 3.5.1. Comparison of cell populations between patient cohorts were done using GraphPad Prism and unpaired t-test with Bonferroni-Dunn correction for multiple comparisons.

### RNAseq pre-processing and analysis

Total RNA quantity and integrity were assessed, using Quant-IT RiboGreen RNA Assay Kit (Invitrogen, Carlsbad, CA, USA) and Agilent Tapestation 2200 RNA Screentape. Purification of mRNA, generation of double stranded cDNA and library construction were performed using TruSeq® Stranded mRNA HT (RS-122-2103) with minor modifications to manufacturer specifications. Minor modifications- the following custom primers (25 µM each) were used for the PCR enrichment step:

Multiplex PCR primer 1.0

5′-AATGATACGGCGACCACCGAGATCTACACTCTTTCCCTACACGACGCTCTTCCGATCT-3′

Index primer

5′-CAAGCAGAAGACGGCATACGAGAT[INDEX]CAGTGACTGGAGTTCAGACGTGTGCTCTTCCGATCT-3′

Indices were according to the eight bases tags developed by WTCHG (Lamble et al.).

Amplified libraries were analyzed for size distribution using the Agilent Tapestation 2200 D1000. Libraries were quantified using Picogreen and relative volumes were pooled accordingly. Sequencing was performed as 75 bp paired-end read on a HiSeq4000 according to Illumina specifications.

The pre-processing of the RNA-seq data was performed through a python 3.6 pipeline using ruffus and cgatcore (https://github.com/cgat-developers/cgat-core/tree/master/cgatcore). For the initial quality control of samples, fastqc was performed on all fastq files and the files were aligned to hg38 using hisat2. We counted the reads using featureCounts and perfomed multiqc on all the results.

DESeq2 apeglm method was used to find differentially expressed genes^66,67^. For PCA plots, we used the variance stabilizing transformation method.

For pathway analysis, we performed fgsea using Hallmark pathways from the MSigDb (Broad Institute). For go-seq analysis, we used the TxDb hg38 database and used significantly differentially expressed genes (*p* < 0.05 and log2FC > 1)^68^.

For Weighted Gene Correlation Analysis^69^, the parameters were softpower = 10, minmodsize = 30, merge tree dissimilarity = 0.25. We exported our results to Cytoscape and used the ClueGO plugin for pathway analysis and visualization.

### Nanostring GeoMx DSP analysis

Sections of 5 μm were cut from FFPE tissue blocks under RNase free conditions, placed onto Leica+ adhesive microscopic slides and subsequently baked over night at 60 °C. Manual slide preparation was conducted according to the NanoString protocol. Briefly, the slide was deparaffinized and rehydrated. Target retrieval was performed using IHC Antigen Retrieval Solution (eBioscience; 10 mM Tris, 1 mM EDTA at pH 9) for 20 min at 100 °C, and then 15 min at 37 °C in 1 μg/ml Ambion Proteinase K (ThermoFisher Scientific). Post-retrieval, the slides were fixed in minutes in 10% neutral buffered formalin and washed. Samples were then treated with UV light (405 nm) for 24 h to quench background autofluorescence. Next, the slide was incubated with human Whole Transcriptome Atlas probes (Nanostring) overnight.

The slide was washed in formamide-SCC buffer before tissue blocking and immunofluorescent staining in Buffer W (Nanostring) with 1% Fc-Receptor block (Miltenyi) and 5% donkey serum (blocking buffer). The sections were then incubated with 1:200 anti-CD68 (SantaCruz, mouse, [KP1]) and 1:200 anti-CD3 (Abcam, rabbit, [SP162]) for 1 h; followed by 1:1000 anti-mouse-AF647, 1:1000 anti-rabbit-Cy3, 1:40 CD45-AF594 (Nanostring) and 1:20,000 Sytox Green for 1 h. All antibody incubations were done in blocking buffer at RT.

The slide was transferred into the Nanosting GeoMx Digital Spatial Profiler for imaging and the manual selection of areas of interest (AOI), from which oligonucleotide probes were collected. For the generation of the library, the samples were subjected to a PCR using i5 and i7 dual indexing primers (Nanostring) before pooling and purification was performed using AMPure XP beads (Beckman Coulter). Quality control of the generated library was done using a Qbit (ThermoFisher Scientific) and TapeStation (Agilent). The resulting library was sequenced on the Illumina NovaSeq platform using 150 bp paired-end sequencing.

The resulting Fastq files were converted into DCC files using the GeoMxNGSPipeline (version 2.0.0.16) on the University of Oxford Advanced Research Computing (ARC) facility^70^ and initial quality control was conducted on the GeoMx DSP analysis suite (version 2.3.0.268). The number of reads during the sequencing steps (>1000 reads), the sequencing saturation (>50%) and the negative probe count geomean (>10) were evaluated against manufacturer recommended thresholds (Supplementary Fig. 8a). Genes which did not rise above the geomean of negative probes+(3*geometric standard deviation) in more than 3 segments or areas of interest (AOI) were excluded, resulting in 15,689 out of 18,677 genes. The filtered transcript dataset was then quantile normalized (preprocessCore 1.58.0) and a principal component analysis was carried out on log2 transformed data (R version 4.2.1, factoextra 1.0.7) (Supplementary Fig. 8b). For cell deconvolution, the package spatialDecon (version 1.6.0) was applied to the normalized data and mapped against cell profiles provided by human adult gut scRNAseq atlas from the Human Cell Atlas project.

### STRING analysis of differentially expressed genes from spatial transcriptomics

The top differentially expressed genes with a *p* < 0.001 between the granuloma region and lamina propria were used as input for STRING database analysis. The top 5 pathways ranked by the false discovery rate for KEGG, Reactome, and Diseases are shown with parameters of a physical subnetwork and a confidence of 0.400.

### Single-cell RNA-sequencing data generation from peripheral blood leukocytes

200 µL fresh whole blood from a single healthy donor were incubated in 2 mL Ammonium-Chloride-Potassium (ACK) lysis buffer in a 50 mL falcon tube for 5 min, washed two times in 1x PBS (Sigma) by centrifugation (800 rpm, 10 min) at room temperature followed by a second round of ACK treatment, washing and spinning. Cells were resuspended in cooled PBS supplemented with 0.5% FCS (Sigma) at 1000 cells/µL. A total of eight samples were processed in parallel. For loading onto the Chromium 10x Genomics platform cells were washed in 1x PBS with 0.04% BSA and resuspended. 10,000 single cells/channel were captured in droplets (less than two hours following phlebotomy).

Library generation for 10x Genomics v2 chemistry was performed following the Chromium Single Cell 3ʹ Reagents Kits User Guide (CG00052). Quantification of libraries was performed using an Agilent Bioanalyzer and Bioanalyzer High Sensitivity DNA Reagents (Cat.# 5067-4627). Single-cell RNA-sequencing libraries were generated using the 10x Genomics Single Cell 3′ Solution (version 2) kit and sequenced to a mean depth of 9981 reads/cell (Illumina HiSeq 4000). An average of 2617 cells/per sample and 913 genes/cell were recovered. Read mapping, quantitation, aggregation of sample count matrices and basic cell filtering (soloCellFilter) was performed using the STARsolo pipeline (star (Version 2.7.5b), samtools (Version 1.9))^71^, version 1.20 (GRCh38) reference sequences, and the Galaxy platform^72^ (Settings: Length of the genomic sequence around annotated junctions = 100, Strandedness of Library = Forward, Collect UMI counts for these genomic features = Gene, UMI deduplication (collapsing) algorithm = All).

A Seurat object was created and counts were filtered to include features detected in at least 3 cells and cells filtered to include at least 200 features using the Seurat R package (v4.0.0)^73^. In addition, data were filtered to exclude droplets for which a high percentage of mitochondrial RNAs (>15%) and for which more than 2500 features (<2500) were detected. Doublet detection and filtering was performed using DoubletFinder (v2.0.3)^74^. Cell cycle stage scoring and annotation was performed by applying the cyclone function implemented in the scran R package (v1.16.0)^75^. The ScTransform function^76^ implemented in the Seurat R package was used to normalize expression values, to scale the data, to identify variable features (﻿variable.features.*n* = 3000), and to regress out mitochondrial features as a source of unwanted variation. Jackstraw permutation tests run on data normalized applying the NormalizeData function to determine significant principal components (*P* < 0.0001). Elbow plots were used to determine the dimensionality of the ScTransfrom-normalized dataset (*n* = 37). The clustree R package (v0.4.3)^77^ was used to produce a visualisation for interrogating clustering over increasing resolutions. The FindClusters function was used to ﻿generate a shared nearest neighbour (SNN) graph and to identify cell clusters (resolution parameter = 1.0). Cell clusters were visualized with the RunTSNE or RunUMAP functions and cluster-specific genes expression profiles were determined using the FindAllMarkers function (Settings: only.pos = TRUE, min.pct = 0.25, logfc.threshold = 0.25). Clusters of cells were assigned identifiers based on manual inspection of differential gene expression profiles and comparison with known cellular gene expression signatures. Dotplots were generated using the DotPlot function in Seurat.

### Publicly available datasets

Publicly available datasets (SCP259, GSE135893, GSE130973) were used for gut, lung, and skin, respectively, to extract the expression of *RAB38, RAB9A, RAB32, HPS1* and *HPS4* expression across different cell types. For the analysis, we used the codes made available by the authors^33,78^. For the gut cells (immune, fibroblast and epithelial) expression levels were scaled to 60%. All the dotplots show average expression and percentage expression across different cell types. HPS1 knockout lung organoid EPCAM + epithelial cells (GSE121999) were FACS sorted to perform RNA-seq and identify changes compared to wild type cells, which we analyzed using the above RNA-seq pipeline^79^.

### Statistical analysis

Data analyses apart from RNA-seq analysis and CyTOF analysis (described above) were performed using GraphPad software (GraphPad software, Inc., San Diego, CA). Statistical tests and significance are mentioned in each figure legend.

### Data accessibility

The bulk RNAseq dataset of HPS1 and control macrophages has been deposited under EGAD00001006978. The single cell data has been deposited at the EGA under EGAS00001005098. The proteomics data have been deposited to the ProteomeXchange Consortium via PRIDE with the identifier PXD024435 and 10.6019/PXD024435.

## Supplementary information

Supplementary Tables

Supplementary Figures
