# Supplementary material for: Hermansky-Pudlak syndrome type 1 causes impaired anti-microbial immunity and inflammation due to dysregulated immunometabolism
Source: Mucosal Immunol. 2022 Oct 27;15(6):1431–46. doi: 10.1038/s41385-022-00572-1 (PMC9607658; doi:10.1038/s41385-022-00572-1)
Supplement: Supplementary file 2 — Supplementary Figures [file 41385_2022_572_MOESM2_ESM.pdf]

Supplementary Figures:

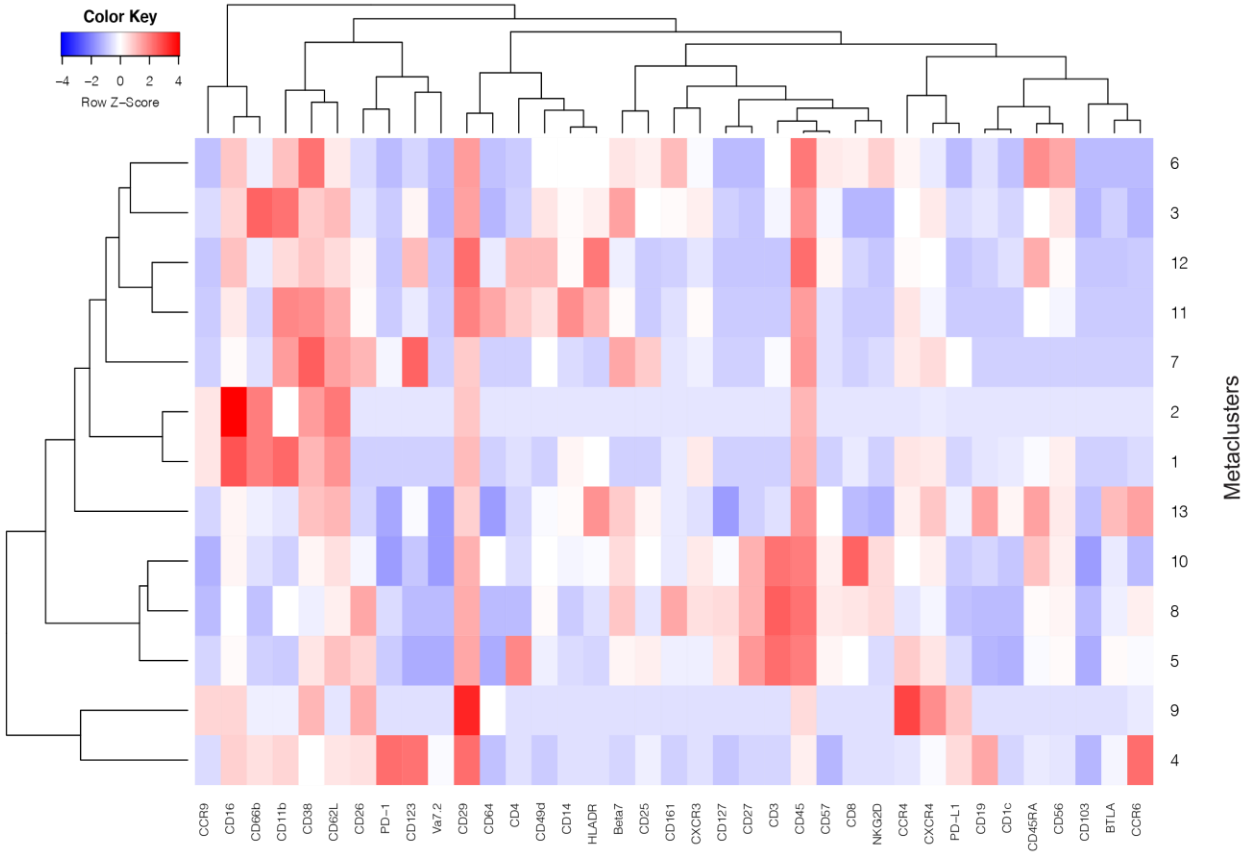

Suppl. Fig. 1. Mass cytometry of peripheral blood surface marker expression.

Heatmap of metaclusters against protein expression to identify corresponding cell types.

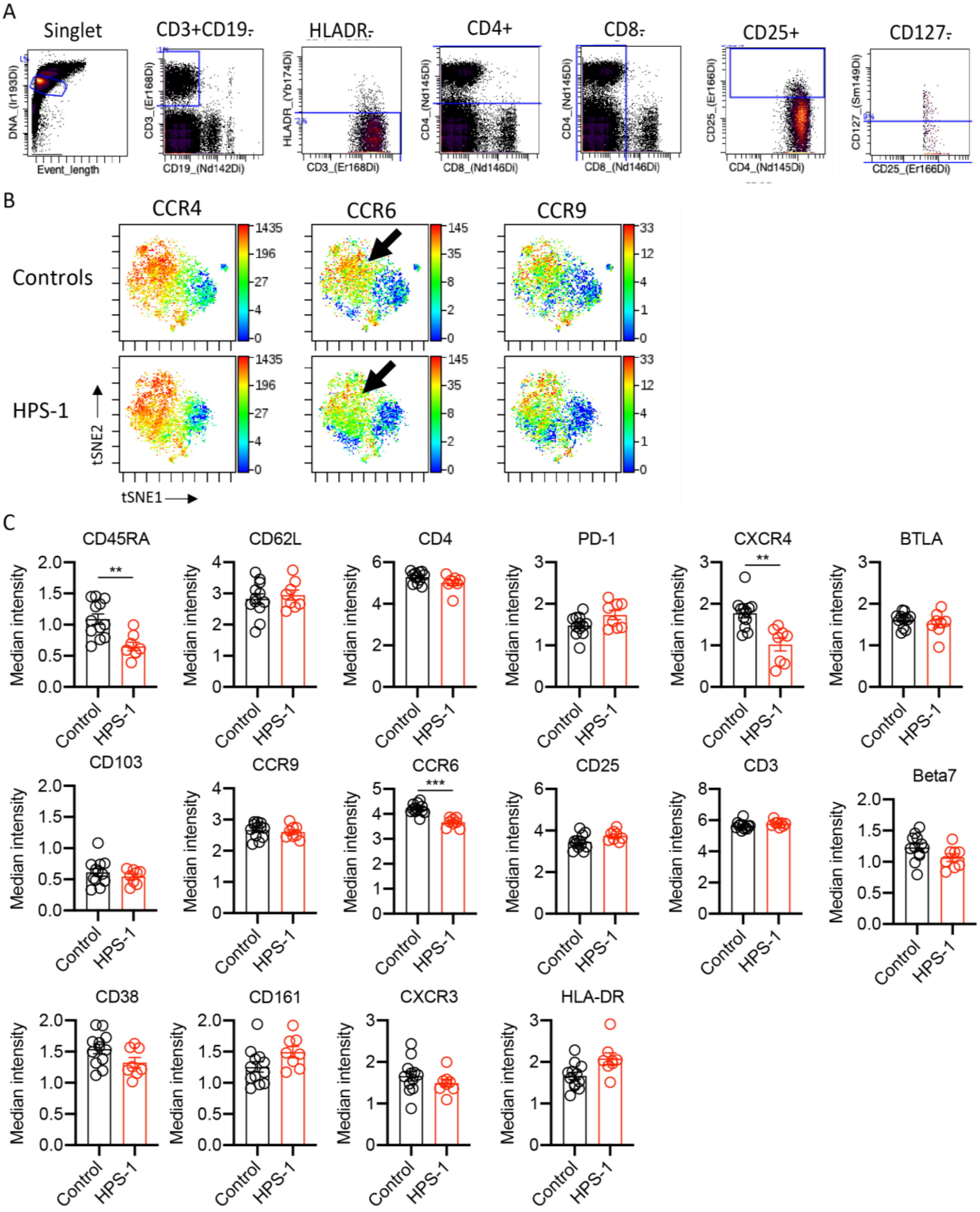

Suppl. Fig. 2. Characteristics of altered T regulatory cell subset in HPS-1 patients compared to controls using mass cytometry. A) Manual gating strategy for T regulatory cells. B) viSNE analysis of manually gated T regs suggests changes in CCR6+ expressing Treg cells (black arrow) consistent with previous FlowSOM analysis. C) The median intensity of proteins for the defined regulatory

T cell cluster is plotted for 12 controls and 8 HPS-1 patients. The Benjamini, Krieger and Yekutieli multiple nonparametric t-test was performed, ** adjusted $p < 0.01$, *** adjusted $p < 0.001$.

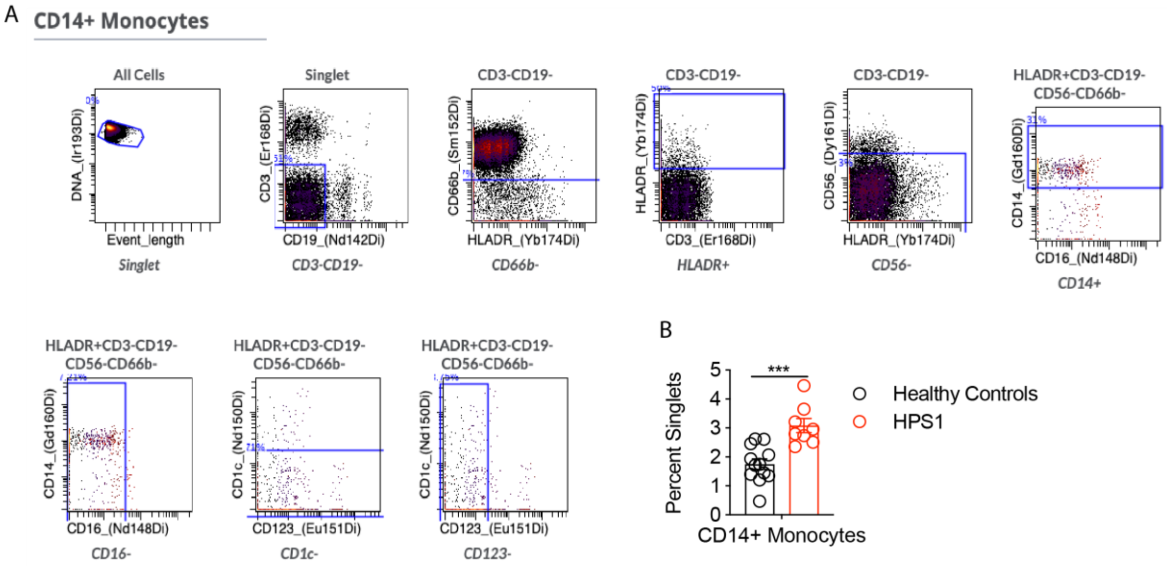

Suppl. Fig. 3. Gating strategy and monocyte enrichment in HPS-1 patient mass cytometry. A) Gating hierarchy B) Comparison of CD14⁺ monocyte abundance in patients with HPS1 or Healthy Controls. Populations are compared with an unpaired t test. Data are mean +/- SEM, *** < 0.001.

A

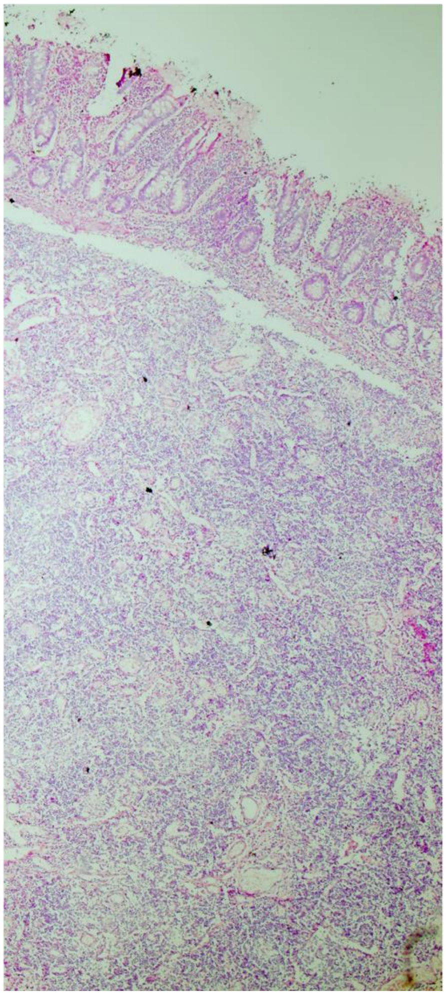

B

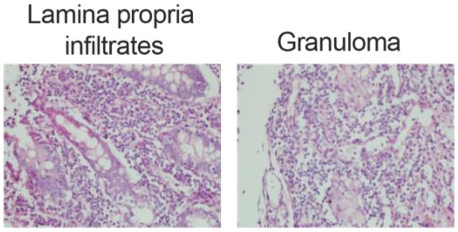

C

Category	term description	observed gene count	background gene count	strength	false discovery rate
KEGG	Lysosome	12	126	1.35	2.19E-10
KEGG	Tuberculosis	7	168	0.99	0.0017
KEGG	Osteoclast differentiation	6	122	1.06	0.002
KEGG	Apoptosis	6	132	1.02	0.0023
KEGG	Phagosome	5	142	0.91	0.0282
Reactome	Neutrophil degranulation	21	473	1.01	2.75E-12
Reactome	Innate Immune System	26	1025	0.77	1.22E-10
Reactome	Immune System	34	1956	0.61	1.96E-10
Reactome	Detoxification of Reactive Oxygen Species	7	37	1.64	3.70E-07
Reactome	Degradation of the extracellular matrix	6	140	1	0.0166
DISEASES	Lipid storage disease	9	78	1.43	5.25E-07
DISEASES	Disease of metabolism	17	997	0.6	0.0014
DISEASES	Disease	47	5921	0.27	0.0014
DISEASES	Inherited metabolic disorder	14	726	0.65	0.0025
DISEASES	Disease of anatomical entity	36	4452	0.27	0.0258

Suppl. Fig. 4. H&E staining of HPS-1 patient gastrointestinal tissue and granuloma pathway analysis. A) Overview image of H&E-stained tissue from a resection of a granulomatous colitis patient. B) Higher magnification images of immune cell infiltrates in the lamina propria and a granuloma. C) STRING analysis of the top differentially expressed genes with a p<0.001 in the granuloma region compared to the lamina propria.

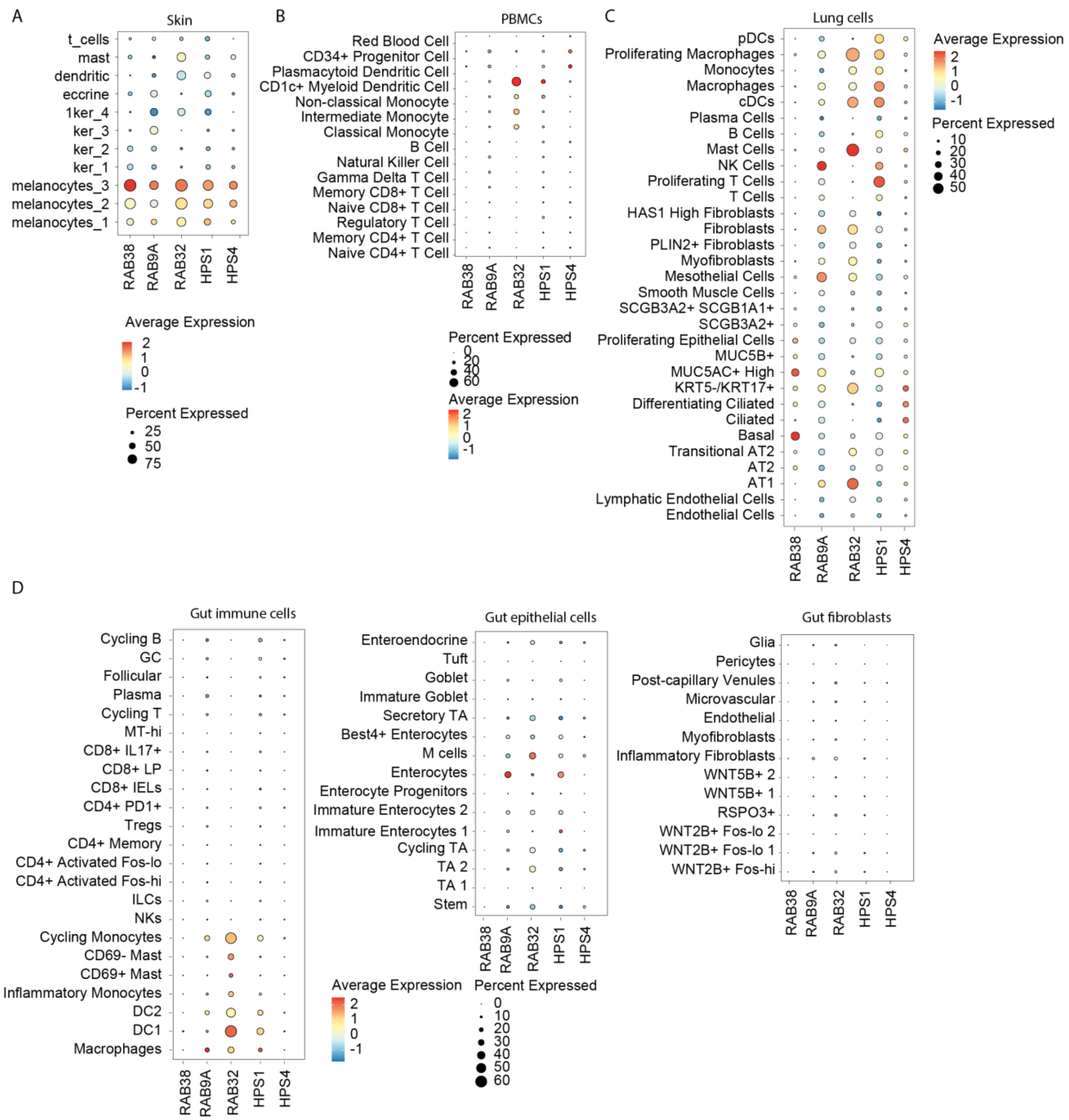

Suppl. Fig. 5. Single cell analysis identifies melanocytes and myeloid cells as expressing the BLOC-3 pathway. A) Single cell analysis of skin biopsies. B) PBMC 10X single cell RNA-seq was performed to visualize BLOC-3 pathway expression across immune cells. C) Lung cell gene expression using an idiopathic pulmonary fibrosis single cell dataset. D) An ulcerative colitis and control dataset was used to visualize BLOC-3 pathway expression in the colon.

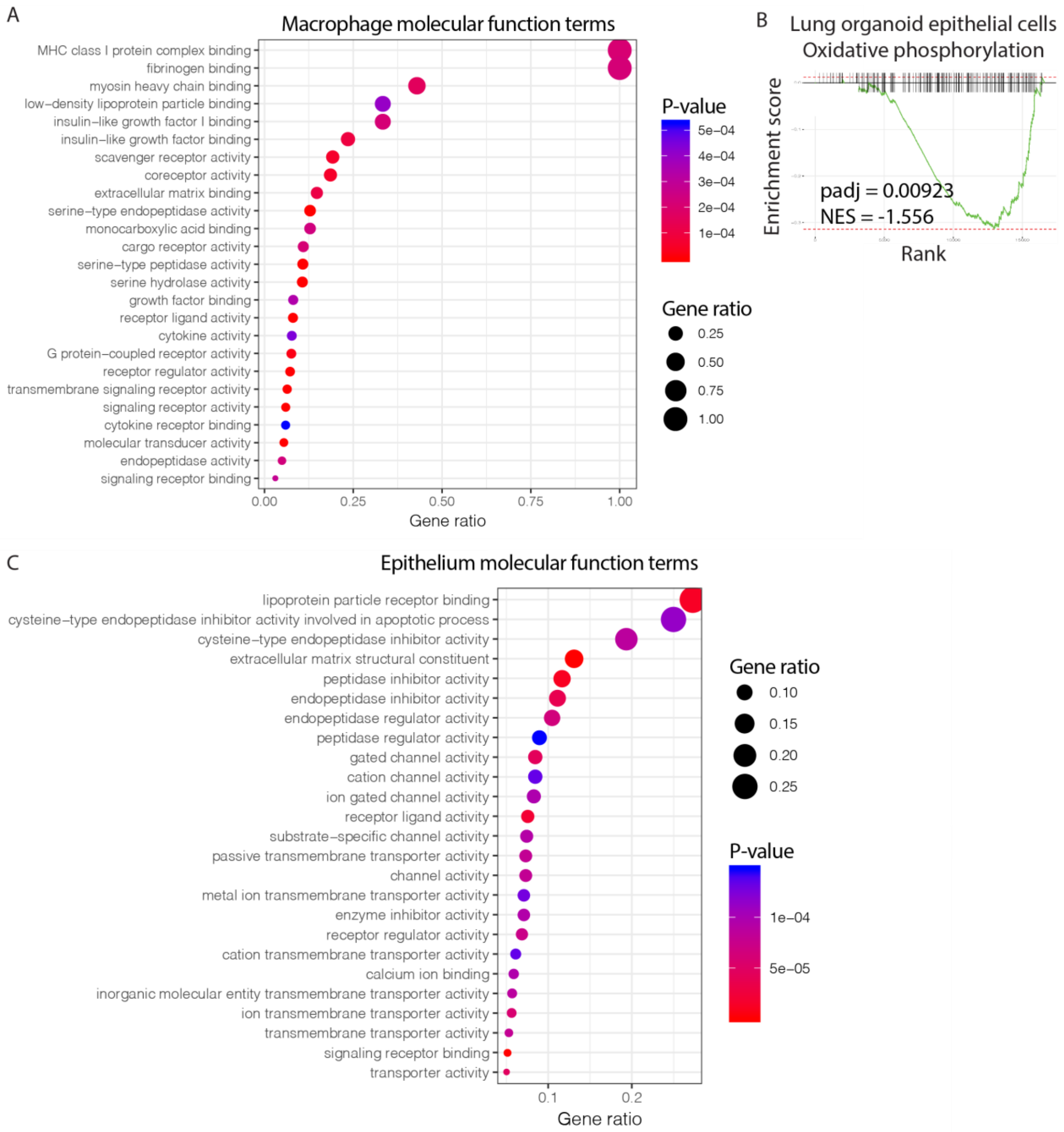

Suppl. Fig. 6. LDL and oxidative phosphorylation altered in HPS-1 macrophages and epithelial cells. A) Gene ontology molecular function terms were used to compare HPS1 and control macrophages. B) Gene set enrichment analysis identifies oxidative phosphorylation as a downregulated module in HPS1 epithelial cells. C) Gene ontology of molecular function terms overrepresented in HPS1-deficient lung organoid epithelial cells. Padj = adjusted p value; NES = normalized enrichment score.

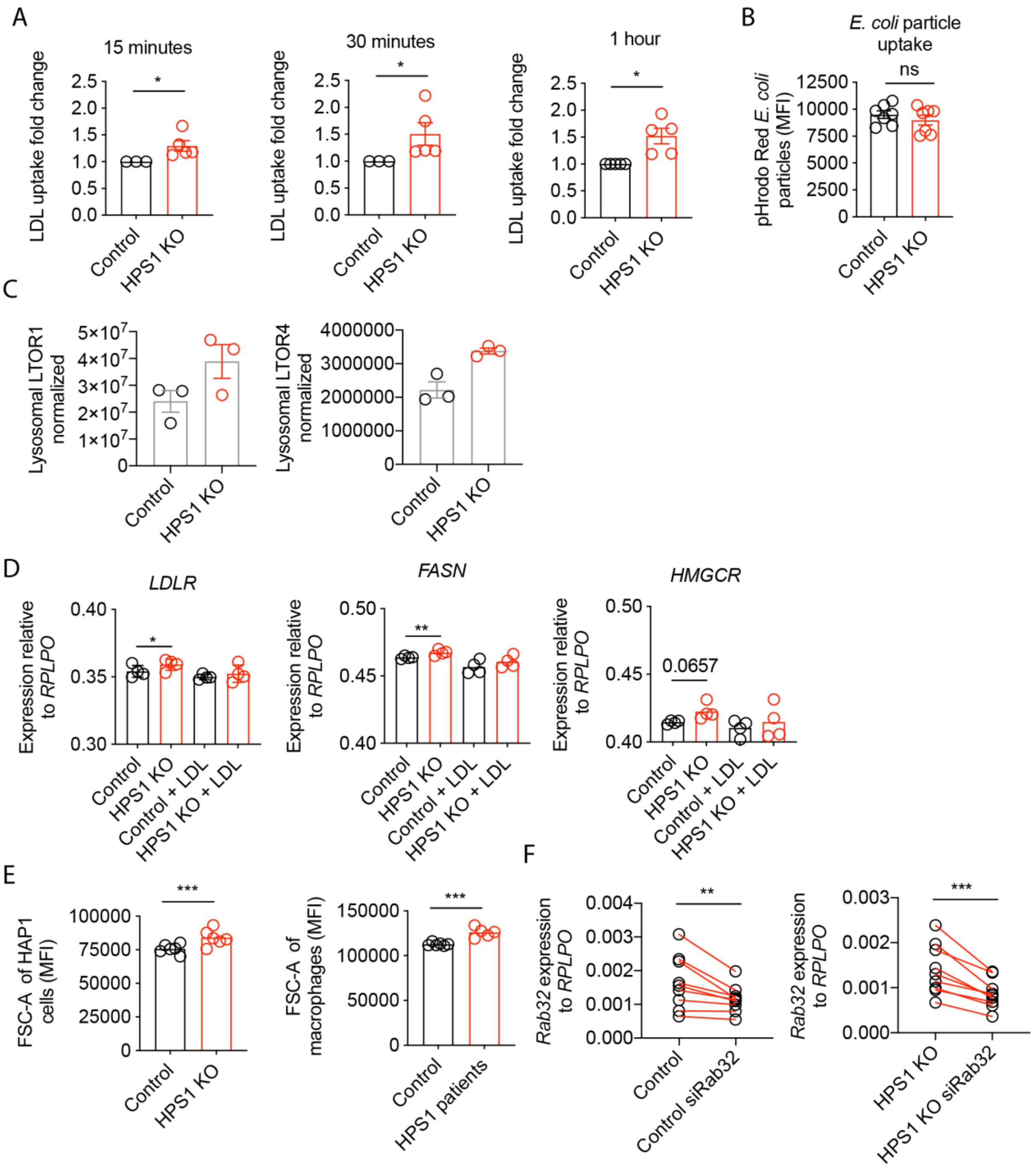

Suppl. Fig. 7. Altered LDL uptake and cell size in HPS1 deficiency. A) Cells were treated with 25 µg/mL LDL for the specified time points (n=5). B) Phagocytosis was measured using *E. coli* particles for 2 hours in HAP1 cells (n=7). C) Lysosomal proteomics abundance of LTOR1 and LTOR4 (n=3). D) HAP1 cells were treated with 2.5 µg/mL LDL for 24 hours and a qPCR was performed (n=4). E) Cell size was determined using the FSC-A mean fluorescence intensity of HAP1 cells (n=6)

and macrophages (n=5). F) A qPCR was performed to examine Rab32 gene expression following Rab32 siRNA treatment (n=9). *$p<0.05$, **$p<0.01$, ***$p<0.001$. For flow cytometry, a ratio paired t-test or an unpaired t-test on log10-transformed data was performed accordingly. For the qPCR, a paired t-test was used.

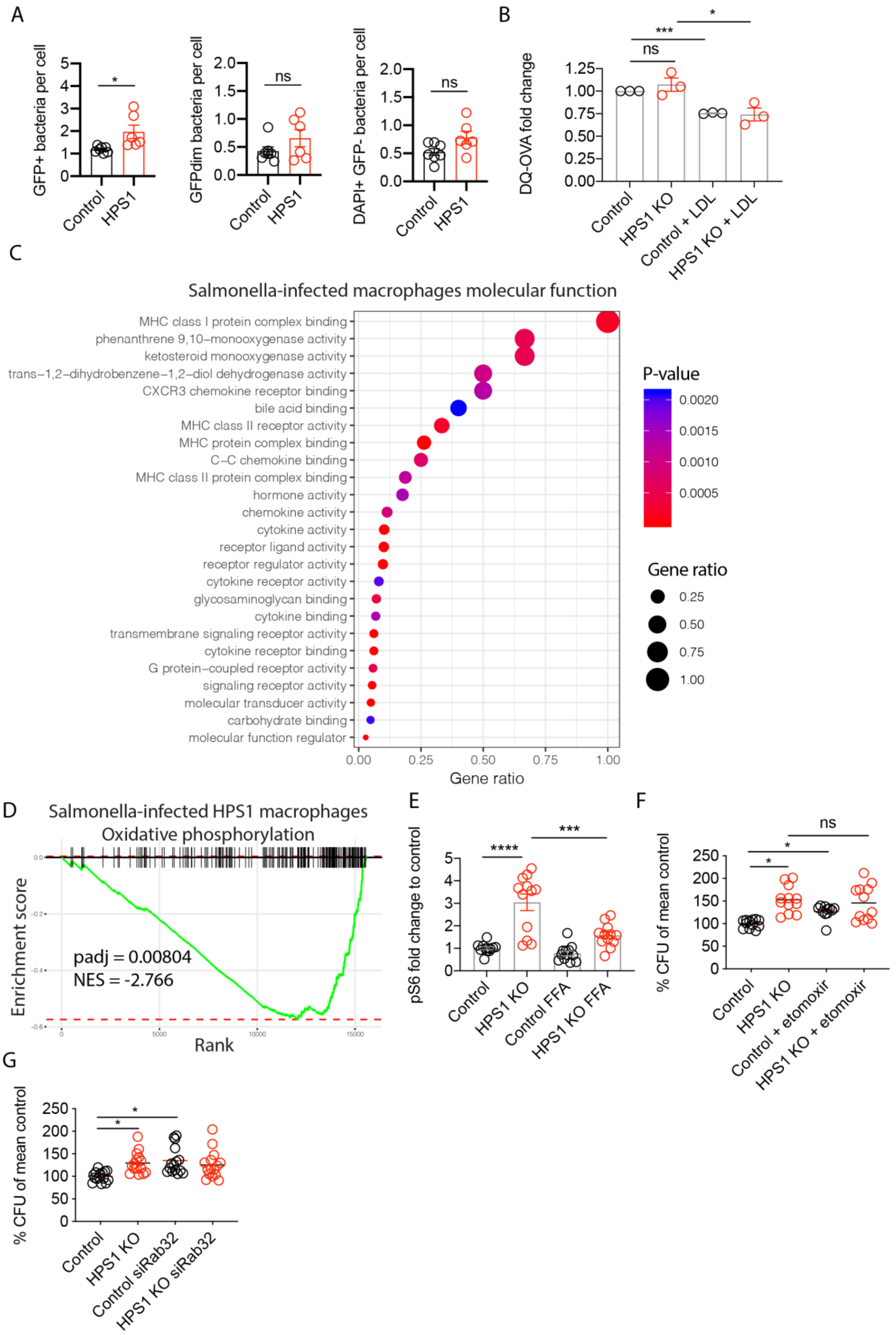

A
GFP+ bacteria per cell
*
Control
HPS1
GFPdim bacteria per cell
ns
DAPI+ GFP- bacteria per cell
B
DQ-OVA fold change
***
*
ns
Control
HPS1 KO
Control + LDL
HPS1 KO + LDL
C
Salmonella-infected macrophages molecular function
MHC class I protein complex binding
phenanthrene 9,10−monooxygenase activity
ketosteroid monooxygenase activity
trans−1,2−dihydrobenzene−1,2−diol dehydrogenase activity
CXCR3 chemokine receptor binding
bile acid binding
MHC class II receptor activity
MHC protein complex binding
C−C chemokine binding
MHC class II protein complex binding
hormone activity
chemokine activity
cytokine activity
receptor ligand activity
receptor regulator activity
cytokine receptor activity
glycosaminoglycan binding
cytokine binding
transmembrane signaling receptor activity
cytokine receptor binding
G protein−coupled receptor activity
signaling receptor activity
molecular transducer activity
carbohydrate binding
molecular function regulator
Gene ratio
P-value
Gene ratio
D
Salmonella-infected HPS1 macrophages
Oxidative phosphorylation
Enrichment score
padj = 0.00804
NES = -2.766
Rank
E
pS6 fold change to control
****
***
Control
HPS1 KO
Control FFA
HPS1 KO FFA
F
% CFU of mean control
ns
*
*
Control
HPS1 KO
Control + etomoxir
HPS1 KO + etomoxir
G
% CFU of mean control
*
*
Control
HPS1 KO
Control siRab32
HPS1 KO siRab32

Suppl. Fig. 8. Impaired bacterial clearance in HPS1 deficiency. A) HPS-1 patient macrophages and control macrophages were infected with GFP-*Salmonella* Typhimurium and microscopy was performed (7 controls, 6 HPS-1 patients). B) HAP1 cells were treated with 25 µg/mL LDL and 10 µg/mL DQ-OVA was used to interrogate lysosomal function (n=3). C) Gene ontology molecular function terms were used to compare HPS-1 and control macrophages after *Salmonella* Typhimurium infection. D) Gene set enrichment analysis identifies oxidative phosphorylation as a downregulated module in HPS-1 patient macrophages following infection. E) HAP1 cells were treated with FFA for 2 hours and stained for S6 phosphorylation (n=6). F) Gentamicin protection assay following 50 µM etomoxir treatment for 16 hours (n=4). G) HAP1 cells were transfected with siRNA against Rab32 or a control pool 72 hours before the gentamicin protection assay (n=5). For flow cytometry and the gentamicin protection assay, a ratio paired t-test was performed; for microscopy data an unpaired t-test was performed *p<0.05, ***p<0.001, ****p<0.0001.

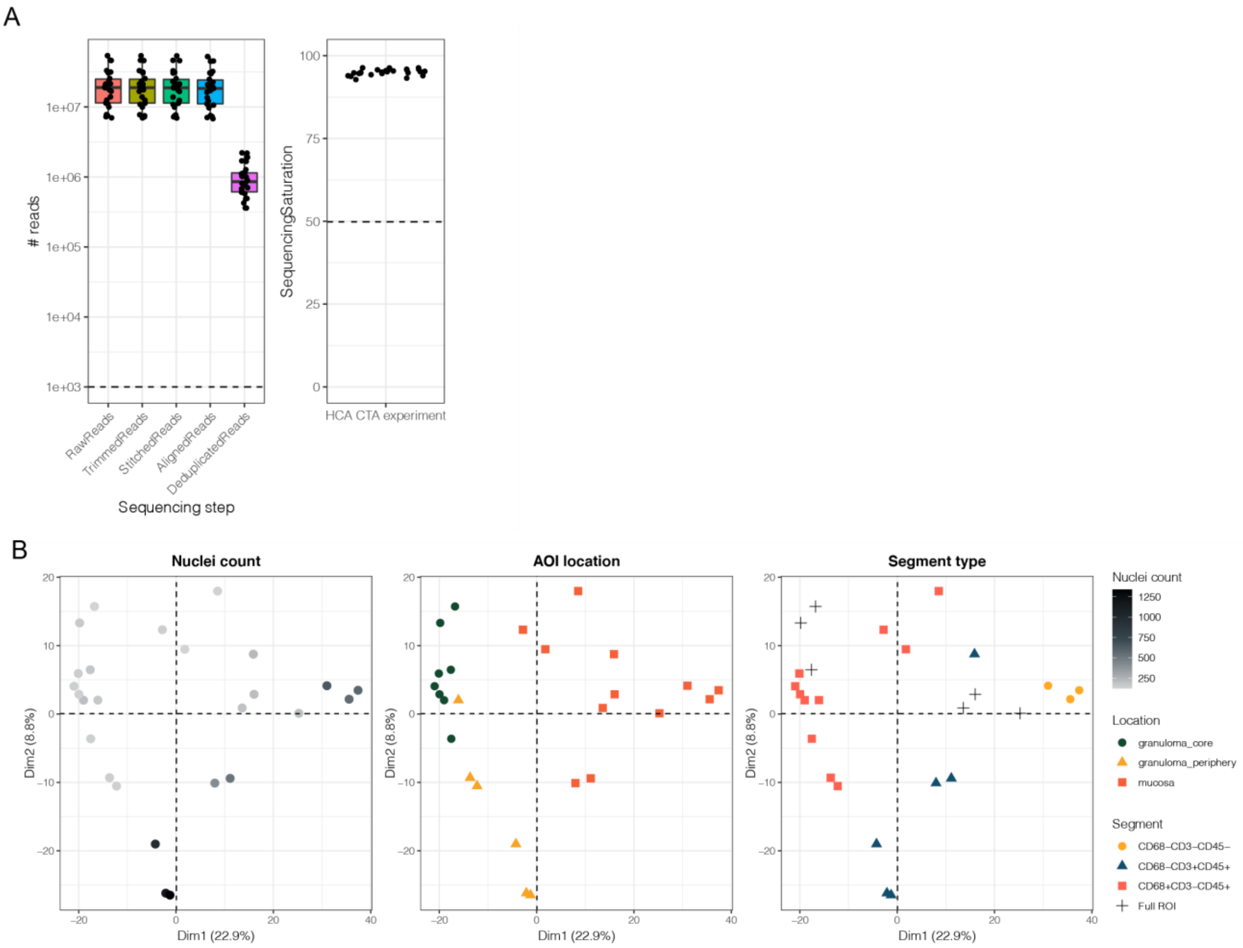

Suppl. Fig. 9. Quality control, normalization and variance of transcriptomic data across areas of interest. A) Consistent read counts across sequencing steps and sequencing saturation are shown for each area of interest (AOI). B) Principal component analysis of the variance between the AOIs. PCA performed on quantile normalized, log2 transformed gene expression values. First and second principal components are plotted and annotated by surface area, location within the slide and segment type.
